# Catsnap: a user‐friendly algorithm for determining the conservation of protein variants reveals extensive parallelisms in the evolution of alternative splicing

**DOI:** 10.1111/nph.18799

**Published:** 2023-02-28

**Authors:** Ksenia Timofeyenko, Dzmitry Kanavalau, Panagiotis Alexiou, Maria Kalyna, Kamil Růžička

**Affiliations:** ^1^ Laboratory of Hormonal Regulations in Plants, Institute of Experimental Botany Czech Academy of Sciences 165 02 Prague 6 Czech Republic; ^2^ Functional Genomics and Proteomics of Plants and National Centre for Biomolecular Research Masaryk University 625 00 Brno Czech Republic; ^3^ Na Vršku 15 150 00 Prague 5 Czech Republic; ^4^ Central European Institute of Technology Masaryk University 625 00 Brno Czech Republic; ^5^ Department of Applied Genetics and Cell Biology, Institute of Molecular Plant Biology University of Natural Resources and Life Sciences (BOKU) 1190 Vienna Austria

**Keywords:** alternative splicing, bioinformatics, determinism, isoforms, machine learning, molecular evolution, transcriptome

## Abstract

- Understanding the evolutionary conservation of complex eukaryotic transcriptomes significantly illuminates the physiological relevance of alternative splicing (AS). Examining the evolutionary depth of a given AS event with ordinary homology searches is generally challenging and time‐consuming.
- Here, we present Catsnap, an algorithmic pipeline for assessing the conservation of putative protein isoforms generated by AS. It employs a machine learning approach following a database search with the provided pair of protein sequences.
- We used the Catsnap algorithm for analyzing the conservation of emerging experimentally characterized alternative proteins from plants and animals. Indeed, most of them are conserved among other species. Catsnap can detect the conserved functional protein isoforms regardless of the AS type by which they are generated. Notably, we found that while the primary amino acid sequence is maintained, the type of AS determining the inclusion or exclusion of protein regions varies throughout plant phylogenetic lineages in these proteins. We also document that this phenomenon is less seen among animals.
- In sum, our algorithm highlights the presence of unexpectedly frequent hotspots where protein isoforms recurrently arise to carry physiologically relevant functions. The user web interface is available at https://catsnap.cesnet.cz/.

## Introduction

In plants, animals, and other eukaryotes, alternative splicing (AS) enables the generation of multiple different mRNAs from a single gene. It is typically a major transcript that codes for a reference (canonical) protein isoform and at least one, generally less abundant, alternative splice variant. Previous studies from diverse organisms have demonstrated that AS can change properties of the resulting proteins (Stamm *et al*., 2005; Kelemen *et al*., 2013; Staiger & Brown, 2013; Szakonyi & Duque, 2018; Chaudhary *et al*., 2019; Kashkan *et al*., 2022b). A significant part of the alternative transcripts are not translated and/or are functionally neutral (Pan *et al*., 2006; Zhang *et al*., 2015; Tress *et al*., 2017). However, many of them carry out relevant regulatory roles, such as control of the protein abundance via coupling with nonsense‐mediated decay (NMD) or by the timing of protein production through nuclear retention of not fully processed transcripts (Lewis *et al*., 2003; Marquez *et al*., 2012; Wegener & Müller‐McNicoll, 2018). Hence, the biological purpose of the most AS events is obscure.

Among the main indicators of the presumed biological relevance is the evolutionary conservation of the AS event (Wang & Brendel, 2006; Keren *et al*., 2010; Tress *et al*., 2017). Combined transcriptomics and computational approaches have been employed to assess the conservation of AS in animals (Modrek & Lee, 2003; Barbosa‐Morais *et al*., 2012; Merkin *et al*., 2012; Xiong *et al*., 2018) and in plants (Wang & Brendel, 2006; Severing *et al*., 2009; Chamala *et al*., 2015; Ling *et al*., 2019). However, the published data frequently show several methodological limitations. For example, almost all of the computer pipelines were designed for the identification of the conserved nucleotides flanking the area modified by the AS event and on the premise that the conserved splice isoforms are encoded by the same AS event type during evolution (Wang & Brendel, 2006; Baek *et al*., 2008; Wang *et al*., 2008; Darracq & Adams, 2013; Xu *et al*., 2014; Chamala *et al*., 2015; Mei *et al*., 2017; Ling *et al*., 2019). Furthermore, these reports have typically centered on a small number of representative organisms (up to 10), omitting the growing complexity of information currently available in public databases (Barbosa‐Morais *et al*., 2012; Chamala *et al*., 2015; Mei *et al*., 2017). In addition, a user‐friendly interface determining the conservation of the provided splice isoforms is lacking. Assessing the conservation of the AS event of interest by a plain Blast search is relatively tricky, owing to challenges in interpreting the data output. Therefore, a simple tool for performing such a task is among the experimental community highly desired.

Machine learning (ML) algorithms gained use as a powerful instrument for solving many biological questions where the given patterns can be learned on a training data set and applied to studied data, including identification of DNA and RNA protein binding motifs and prediction of splice sites (Zitnik *et al*., 2019). Logistic regression is among the most efficient classification algorithms in ML, which achieves remarkable performance in binary classification (Lever *et al*., 2016; Subasi, 2020).

Here we present the Catsnap pipeline (Conserved AlTernative SpliciNg in Animals and Plants). It employs a logistic regression ML model to assess the conservation of protein isoforms, comparing them to those deposited in RefSeq and GenBank. The algorithm does not take into account the type of AS event. Therefore, it is also well suited for detecting the instances where the AS events evolved several times independently with a repetitive tendency to impact equivalent protein features. A web interface dedicated to a common user is available at https://catsnap.cesnet.cz/.

## Materials and Methods

### Sequence database

The internal Catsnap database was made by gathering protein sequences originating from genes with at least one AS event, alternative transcription start site (AltTSS) or alternative cleavage and polyadenylation in their coding regions. They were obtained from the curated RefSeq database (release 204, January 4, 2021), and extended with the complementary GenBank data set for plants (release 242.0, February 16, 2021). The full‐size Catsnap database contains sequences from 176 plants and 701 animals, and the reduced database includes 176 plant and 97 animal species (Supporting Information Table S1).

### Processing of query sequences without a database accession number

For the cases when a protein sequence of interest is not present in RefSeq (animals) or RefSeq and Genbank (plants) databases, we introduced the possibility of testing the sequences provided by the user. The algorithm requires *nucleotide* coding sequences of both isoforms (lacking the untranslated regions) and the sequence of the gene of their origin (unspliced transcript). For the user's comfort, Catsnap removes any character different from A, T, G, C. Then, the three entered sequences are aligned by Muscle (Edgar, 2004) to determine the exon–intron structure, and the coding regions of the alternative isoforms are translated to amino acids. The genetic code is translated using the Standard codon table from Biopython Project (Cock *et al*., 2009).

### ML model, training set, and features

Initially, 31 conserved protein pairs from *Arabidopsis thaliana* (L.) Heynh. were selected from the literature (Table S2). They were Blasted against the RefSeq database of plant proteins. The obtained sequences were ordered in pairs most similar to the query canonical and alternative isoforms, respectively, using the Mega multiple sequence alignment software (Kumar *et al*., 2018). Then, the 31 arrays containing a total of 1426 isoform pairs were used as an ML training set. To evaluate the model performance and prevent overfitting, we performed cross‐validation by sequentially excluding each of the 31 initial protein pairs and all its filial hits from the training set. A logistic regression ML model from the Scikit library (linear_model.LogisticRegression) (Pedregosa *et al*., 2011) was employed. Four independent ML features were specified for the ML model (Fig. S1):

1. The amino acid sequence similarity was set as a bit score provided by the Blast alignment. It favors sequence pairs, which show the highest overall sequence similarity with those entered as a query.
2. The mutual exclusivity of the regions affected by AS (AS regions) in the sequence pair is calculated according to the formula: *F*_2_ = |*Q* Δ *H*|/|*Q*| + |*H*|, where *Q* and *H* are sets of positions of aligned AS regions of the query and hit sequences, respectively, and |*Q* Δ *H*| is the symmetric difference between query and hit in the AS region (Fig. S1a,b).
3. Amino acid similarity of AS regions is the number of matching amino acids in the AS regions in the query and hit within the subregion determined by Blast as matching (*m*), divided by the length of this whole subregion (*l*) (*F*_3_ = *m*/*l*). This feature particularly weights short conserved sequences. Identical subregions will get a score equal to 1 (Fig. S1c).
4. Amino acid dissimilarity shows the proportion of the matching amino acids (*m*) identified by the feature (3) in the context of the length of both AS regions. The formula for this feature is *F*_4_ = (*q* + *h* − 2*m*)/(*q* + *h*), where *q* is the length of the AS region of the query, *h* is the length of the AS region in the hit sequence, and *m* is the number of matches in the matching subregion. Identical subregions will get a score equal to 0 (Fig. S1d).

On the basis of the listed features, the sequence pairs receive a similarity score, which reflects the closeness of the hit pair to the query pair. The similarity score ranges from 0 to 1, where 1 is complete identity. The score is used to sort the output list of identified hits from the most similar to the least similar. For the isoforms having more than one AS region, each AS region obtains a location identifier describing its position relative to the regions processed by constitutive splicing by counting the number of the uninterrupted constitutively spliced regions from the N‐ and C‐ terminal direction (exemplified on the Fig. S1e).

The code of the algorithm is available at GitHub (https://github.com/kdcd/catsnap).

## Results

### Catsnap – an ML computational pipeline for the identification of conserved AS

**Fig. 1:**
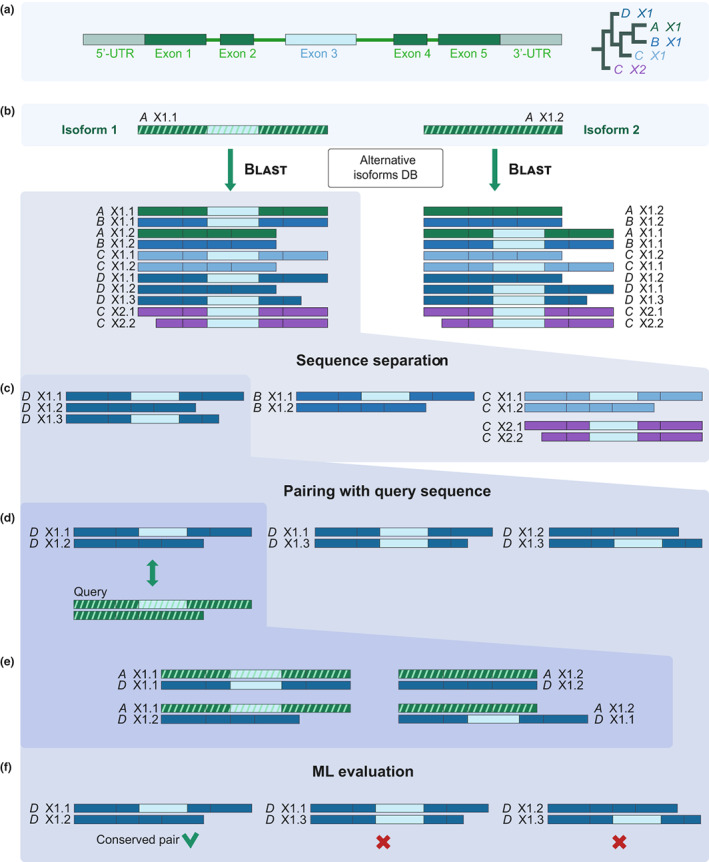
The scheme of the Catsnap algorithmic pipeline. (a) A diagram of an example gene *X1* with alternative skipping of exon 3 (turquoise); the outline of its imaginary phylogenetic relationships (from Organism *A* to *D*) is on the right. (b) The query isoforms *A* X1.1 and *A* X1.2 (hatched) are Blasted against the internal database of protein isoforms. The Blast output also contains isoforms originating from unrelated alternative splicing (AS) events (*D* X1.3) or occurring in paralogous genes (*C* X2). (c) The protein isoforms identified by Blast are separated according to the gene name and organismal source. (d) Protein isoforms from each organism are paired by all combinations. (e) Each generated pair is re‐associated with the query sequences using initial Blast alignments (c), and, (f) evaluated.

For assessing the conservation of a pair of isoforms of interest, the Catsnap pipeline analyzes two protein queries. Isoform 1 is typically the reference (canonical), and Isoform 2 alternative isoform (Fig. 1a,b). They can be provided as RefSeq accession numbers or as nucleotide sequences. In the first step, both sequences are separately Blasted against the internal database of protein variants (see the Materials and Methods section; Fig. 1b). As this is the most computationally demanding step of the pipeline, the user can select whether the full‐sized or reduced (Table S1) database will be searched. Both Blast output lists usually contain, besides the companion sequence and homologous isoforms, also those resulting from unrelated AS events (Fig. 1b, *D* X1.3) or AS events of paralogous genes (Fig. 1b, *C* X2.1 and *C* X2.2). Next, using the RefSeq gene annotation, the algorithm separates the sequences assigned to each species and gene (Fig. 1c) and creates all possible pairwise combinations within these subsets (Fig. 1d).

To determine which of the rearranged sequence pairs are similar to the query (i.e. a conserved protein pair), the logistic regression ML model is employed. It uses pairwise alignments and scores provided by Blast (Fig. 1e,f). The following ML features have been implemented: whole sequence amino acid similarity as determined by Blast, (2) the position of the (non‐)aligned amino acids within the AS region; and features (3) and (4) which score amino acid similarity specifically within the AS region (Fig. S1; see the Materials and Methods section). The candidate orthologous isoforms are returned as a file in the Fasta format, sorted from the highest to the lowest score. A large number of sequences found can complicate a quick visual examination of the results. Therefore, a list containing the single most similar isoform pair per species is also available for download and can be directly analyzed online by a built‐in Muscle alignment tool (Fig. S2).

### Plants tend to show a high degree of plasticity of the AS types during evolution

**Fig. 2:**
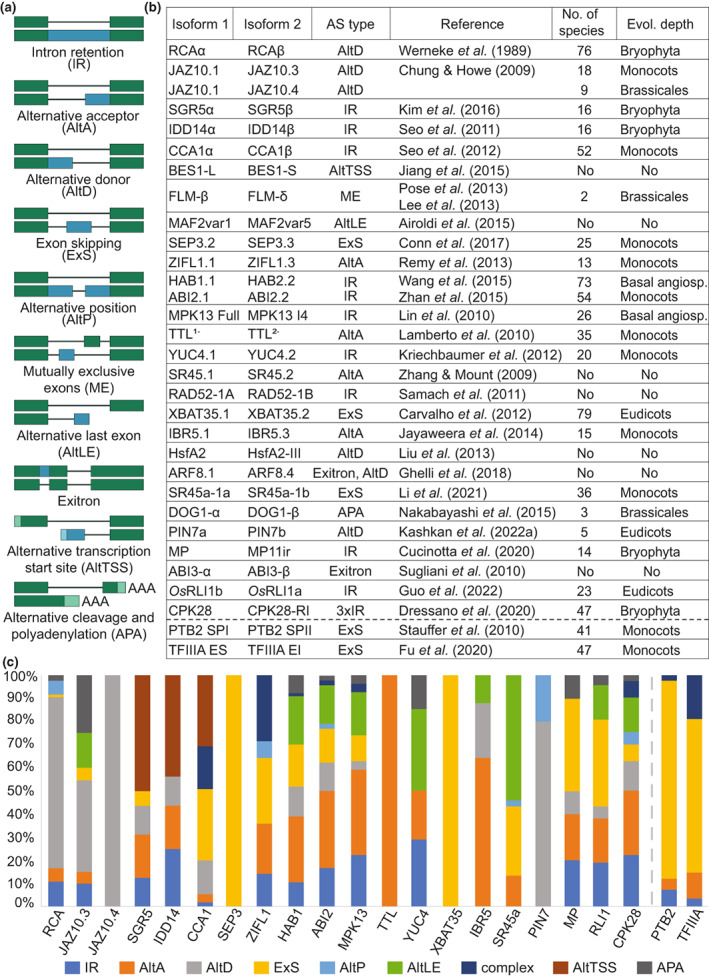
Evolutionary conservation of functionally validated alternative isoforms from plants. (a) The schemes of relevant alternative splicing (AS) types, in addition to the alternative transcription start site (AltTSS) and alternative polyadenylation (APA). Dark green and dark blue colors represent coding constitutive and alternative regions, respectively; light green and light blue colors are for non‐coding constitutive and alternative regions, respectively. (b) The conservation of validated *Arabidopsis thaliana* and *Oryza sativa* (*Os*) isoforms within main plant phylogenetic lineages, including example transcripts without protein‐coding potential (undergoing nonsense‐mediated decay (NMD), separated by dashed line). (c) In most of the functionally validated plant AS events, the changes seen at the amino acid level are widely conserved, independent of the AS type (or AltTSS and APA), it even includes the transcripts undergoing NMD (separated by dashed line); the *y*‐axis denotes the proportions of alternative isoforms arising from the different outlined mRNA processing type.

To validate the outlined algorithmic pipeline, we examined the depth of conservation of prominent experimentally validated protein isoforms from plants (Staiger & Brown, 2013; Brown *et al*., 2015; Hrtyan *et al*., 2015; Shang *et al*., 2017; Szakonyi & Duque, 2018; Kashkan *et al*., 2022b; Figs 2, S3a; Table S3). Those identified in *Arabidopsis* generally showed evolutionary conservation within Brassicales or deeper, as evidenced by a number of hits from the RefSeq and GenBank databases. This underlines that, indeed, the majority of functionally relevant protein isoforms tend to be sustained during evolution and that Catsnap provides a reliable baseline for assessing their conservation.

The Catsnap pipeline is designed to assess the conservation of AS with the outcome at the protein level (Fig. 1). For example, two *Arabidopsis* isoforms of TRANSTHYRETIN‐LIKE PROTEIN (TTL) differ in the presence of a peroxisome targeting signal, whose inclusion is regulated by the alternative acceptor site (AltA) in the third intron (Lamberto *et al*., 2010). Catsnap finds the predicted peroxisome‐ and cytosol‐targeted isoforms in 35 eudicot and monocot species. However, in *Glycine max, Solanum lycopersicum*, AltA removes sole glutamate and does not affect the predicted peroxisome targeting signal, evidencing that this event is likely non‐homologous in these species (Fig. S4). Hence, the evolutionary history of this event documented by Castnap illustrates the relevance of determining the conservation of AS events at the amino acid level.

From the principle, Catsnap is able to identify the instances where the AS type varies, but the homology of the resulting amino acid sequence persists. We searched in the literature whether there were such examples presented earlier. AS of *RUBISCO ACTIVASE* (*RCA*) was among the first AS events identified in plants (Werneke *et al*., 1989; Reddy, 2007). The resulting longer RCAα and shorter RCAβ isoforms show a differential ability to activate the Rubisco enzyme (Zhang & Portis, 1999; Zhang *et al*., 2002). Both proteins have been detected in multiple species, including *Arabidopsis* or various monocots (Salvucci *et al*., 1987). To *et al*. (1999) characterized the RCA isoforms in rice. They indeed noticed that the isoforms result from a different AS type than in other species, including *Arabidopsis*. The Catsnap algorithm, in accord with the focused *RCA* event evolutionary analysis (Nagarajan & Gill, 2018), revealed that the RCA isoforms are produced even by more AS types in various plants (Fig. S5). It illustrates that the protein isoforms can be functionally conserved and show high plasticity of AS types they arise from.

*JASMONATE‐ZIM‐DOMAIN PROTEIN 10* (*JAZ10*) produces, besides the canonical JAZ10.1 isoform, a frame‐shifted JAZ10.4 protein, which interferes with the protein interactions required for JAZ10.1 signaling. This gene also codes for the JAZ10.3 protein (from two transcripts), which impedes the JAZ10 signaling pathway in a moderate way (Chung & Howe, 2009; Moreno *et al*., 2013; Fig. S6a). Catsnap detects the JAZ10.4 orthologs in multiple species within the Brassicaceae family, being the products of the same AS type. JAZ10.3 orthologs are found in eudicots and monocots. However, the Catsnap search revealed that the types of AS in *JAZ10.3* highly vary among other plants (Figs 2a, S6b,c). Moreover, SGR5β, a truncated non‐DNA binding isoform of the transcriptional factor SHOOT GRAVITROPISM 5 (SGR5) (Kim *et al*., 2016), can be seen in the wide range of plant species, including the liverwort *Marchantia polymorpha*. We observed, too, that the different AS types and AltTSSs lead to the production of the proteins matching the alternative SGR5β isoform (Fig. S7). Thus, the Catsnap pipeline efficiently allows for detecting the instances when the isoforms are functionally conserved at the amino acid level, but underlying mechanisms at the nucleotide level (AS types or AltTSS) can differ during gene evolution.

*CALCIUM‐DEPENDENT PROTEIN KINASE 28* (*CPK28*) produces an alternative isoform with a premature termination codon (PTC), predicted to remove Ca^2+^‐binding EF‐hands domains, required for the kinase activity of the resulting protein (Dressano *et al*., 2020). This is likely a characteristic of the broader CPK family (CDPK) and was suggested as exerted via several AS types among angiosperms (Loranger *et al*., 2021). Accordingly, Catsnap recognizes the conservation of the shortened CPK28‐RI isoforms up to bryophytes (Fig. S8). Interestingly, the presented molecular model (Dressano *et al*., 2020) does not exclude, in principle, the scenario that the *CPK28‐RI* transcript might actually not be translated. Hence, we also examined the transcripts, which have earlier been shown to be controlled by AS coupled with NMD. They are associated with PTCs; they are not translated and undergo subsequent degradation (Lewis *et al*., 2003). Among them, the NMD‐controlled autoregulatory circuits of the polypyrimidine tract‐binding proteins (PTBs) have been intensively investigated (Wollerton *et al*., 2004; Stauffer *et al*., 2010). Indeed, the sequences related to the imaginary truncated protein derived from the *Arabidopsis* alternative *PTB2 SPII* transcript were found in 41 plant species up to monocots (Fig. S9). Similarly, the expression of *TRANSCRIPTION FACTOR FOR POLYMERASE III A* (*TFIIIA*) is autoregulated via the generation of an alternative NMD‐dependent transcript (Fu *et al*., 2009). Catsnap finds the nominal shortened proteins in 25 angiosperms (Fig. S10). Thus, Catsnap is able to detect the conservation of a regulatory event (even) regardless of the presumed isoform translatability.

As it appears that many plant genes show a high degree of plasticity of the AS types during evolution, we systematically inspected the types of AS in the protein sequences related to the remaining experimentally validated AS events in plants (Fig. 2b). Strikingly, we found that the variability of AS types is seen among the most well‐characterized alternative isoforms (Fig. 2c). Altogether, we conclude that examining the evolutionary conservation on the basis of amino acid sequence, as provided by Catsnap, brings a remarkable insight into the evolutionary conservation of AS. The homologous protein isoforms are maintained during evolution, but the underlying AS types can largely vary with respect to the organismal group.

### The evolution and plasticity of AS in animals

**Fig. 3:**
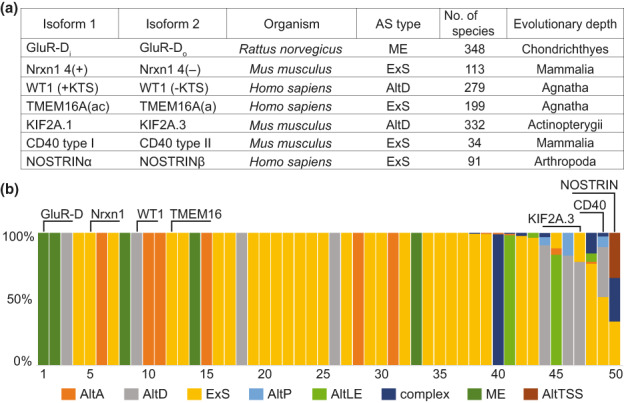
Conservation of functionally validated alternative isoforms from various animal model organisms. (a) The summary of the evolutionary history of validated animal protein isoforms discussed in the main text. (b) Some validated alternative splicing (AS) events in animals show evolutionary plasticity; several detected rare changes of AS type can be perhaps ascribed to the misannotation of the corresponding transcript. The values on the *x*‐axis indicate the analyzed isoform pairs listed in Supporting Information Table S4; the *y*‐axis denotes the proportions of alternative isoforms arising from different AS types, alternative transcription start site (AltTSS) and alternative polyadenylation (APA).

To test the versatility of the Catsnap pipeline further, we also examined the conservation of prominent characterized AS events in animals (Stamm *et al*., 2005; Kelemen *et al*., 2013). For example, for the GluR‐D_i_ and GluR‐D_o_ isoforms of the AMPA‐type ionotropic glutamate receptors (Sommer *et al*., 1990; Mosbacher *et al*., 1994; Dawe *et al*., 2019; Zhao *et al*., 2019), we found that both are conserved among > 300 vertebrate species ranging from the cartilage fishes (Chondrichthyes) to mammals (Figs S3b, S11). Furthermore, the Catsnap pipeline documented a stable and long evolutionary history of selected prominent animal events, including that of neurexin I (*Nrxn1*) (Iijima *et al*., 2011), the Wilms tumor susceptibility gene (*WT1*) (Larsson *et al*., 1995), transmembrane 16A (*TMEM16A* or *anoctamin1*) (Ko *et al*., 2020; Fig. 3a), and a large number of other functionally confirmed isoforms (Figs 3b, S3b; Table S4). Altogether, this demonstrates that although the Catsnap ML algorithm has been trained on plant sequences (see the Materials and Methods section), it can also be used for examining the evolutionary history of protein isoforms in other eukaryotes, including animals.

We analyzed the AS plasticity of the experimentally validated AS events from animals, too (Fig. 3b). The KIF2A.3 isoform of the microtubule destabilizer Kinesin family member 2A lacks 20 amino acids by the alternative choice of a donor splice site (AltD) in the 5^th^ intron (Akkaya *et al*., 2021; Figs 3, S12a). The alternative inclusion of the internal protein motif is conserved up to the ancient teleost fishes. However, in the evolutionarily derived bony fishes (Euteleostei), this region is encoded by a separate exon, and the event is regulated by exon skipping (Fig. S12b–d). The alternative isoform of tumor necrosis factor receptor CD40 lacks a C‐terminal part present in the canonical protein, including a transmembrane domain (Tone *et al*., 2000; Eshel *et al*., 2008; Hou *et al*., 2008). The truncated version is conserved in mammals and is processed by multiple AS types as well (Fig. S13). Human NOSTRIN (eNOS trafficking inducer) undergoes shortening of its N‐terminus in the stressed liver, resulting in the NOSTRINβ isoform (Mookerjee *et al*., 2007; Wiesenthal *et al*., 2009). NOSTRINβ is conserved up to cartilaginous fishes and arthropods and arises from exon skipping, various combinations of AS types and AltTSSs (Fig. S14). Taken together, although much less prevalent than in plants (compare Figs 2c, 3b), the evolutionary plasticity of AS types is also seen in animal systems.

## Discussion

Several studies demonstrated that evolutionary conservation closely correlates with the functionality of a given isoform (McCartney *et al*., 2005; Lamberto *et al*., 2010; Astro *et al*., 2022). On the contrary, another set of reports has presented that mutations underlying AS are linked with recent evolutionary adaptations, highlighting the transitivity of AS events (Barbosa‐Morais *et al*., 2012; Ling *et al*., 2019; Wright *et al*., 2022). To address the two extreme viewpoints, as a proof of concept of the algorithm, we show here that the functionally validated isoforms arising from AS tend to exhibit prevailingly deeper evolutionary origin. Moreover, we have designed a user‐friendly interface, available at https://catsnap.cesnet.cz/, which allows, even in a batch mode, for a quick visual overview of the evolutionary conservation of protein (transcript) variants of choice. Together with other available large‐scale data resources (e.g. Berardini *et al*., 2015; Martín *et al*., 2021; Cunningham *et al*., 2022; Gramates *et al*., 2022), it is aimed to help the researcher to hint at whether the event of interest could have a detectable biological function, and be, for instance, suitable for further experimental characterization.

In contrast to the effort done previously (Barbosa‐Morais *et al*., 2012; Merkin *et al*., 2012; Darracq & Adams, 2013; Chamala *et al*., 2015; Ling *et al*., 2019), we employed an amino acid sequence view on the conservation of AS. Our approach reveals that the expression of the conserved functional isoforms is in different plant species likely commonly controlled by different types of AS. It should be underlined that the protein models present in the current databases mostly arise from algorithmic predictions. The annotated proteins may show a different authentic amino acid sequence or might not be translated at all. Thus, the results should be interpreted critically (Brown *et al*., 2015). On the contrary, from previous reports, the two RCA isoforms, processed by multiple types of AS (Figs 2c, S5; Nagarajan & Gill, 2018), have been functionally characterized in different plant species (Werneke *et al*., 1989; To *et al*., 1999; Xu *et al*., 2017). A similar phenomenon has recently been proposed for the isoforms of REGULATOR OF LEAF INCLINATION 1 (RLI1a and b) in rice and *Arabidopsis*, where they show a high evolutionary plasticity of the AS events controlling their expression (Fig. 2c; Guo *et al*., 2022). Moreover, the individual RCA isoforms can even be encoded by separate genes in several species (Salvucci *et al*., 2003; Yin *et al*., 2010; Nagarajan & Gill, 2018) and the differentially localized auxin synthase isoforms YUCCA4 (YUC4) are parallelized by individual gene products of the *YUC* family in *Arabidopsis* (Fig. 2c; Kriechbaumer *et al*., 2012, 2016). Taken together, it seems that particularly plant genes show a high extent of evolutionary plasticity of protein isoforms controlled by AS.

In contrast to the situation in animals, plants show high variability in genome sizes (explicitly in terms of ploidy), general DNA organization and sequence divergence. In addition, their coding and non‐coding sequences evolve faster (Leitch & Leitch, 2008; Kejnovsky *et al*., 2009; Murat *et al*., 2012). Our analysis of the experimentally validated alternative isoforms revealed that the AS patterns in plants broadly vary compared to animals as well. Regulation of AS is jointly carried out by the *cis*‐elements, encoded by the pre‐mRNA sequence, and *trans*‐acting protein regulators, which bind these motifs (Reddy, 2007; Fu & Ares, 2014). Ling *et al*. (2019) noted a considerably high rate of gains and losses of AS among plant transcriptomes analyzed, strongly linked with the rapid evolution of plant *cis*‐elements (Shen *et al*., 2014; Thatcher *et al*., 2014; Ling *et al*., 2019; Wang *et al*., 2019). Altogether, the presented plasticity of the prominent protein isoforms in plants represents just another manifestation of the remarkable variability of their genomes. Hence, the conservation of the protein isoforms can mark a hotspot that leads to the production of the same evolutionary conserved regulators and recurrent functional adaptation.

## Competing interests

None declared.

## Author contributions

KT, DK, PA, MK, and KR conceptualized the research. KT and DK designed the algorithm. KT, MK and KR analyzed the data. KT and KR wrote the manuscript. All authors read and approved the final version of the manuscript.

## Supporting information


**Fig. S1** The outline of the Catsnap machine learning features.
**Fig. S2** The snapshots of the Catnap graphical output interface.
**Fig. S3** Schematic relationships of the main plant and animal phylogenetic groups.
**Fig. S4** Alternative splicing of *TTL* from representative plant species.
**Fig. S5** Alternative splicing of *RCA* in various plants.
**Fig. S6** Alternative splicing of *JAZ10* in various plants.
**Fig. S7** Alternative splicing and alternative transcription start sites of *SGR5* in various plants.
**Fig. S8** Alternative splicing of *CPK28* in various plants.
**Fig. S9** Alternative splicing of *PTB2* in various plants.
**Fig. S10** Alternative splicing of *TFIIIA* in various plants.
**Fig. S11** Alternative splicing of *Glu4* in various animals.
**Fig. S12** Alternative splicing of *Kif2a* in various animals.
**Fig. S13** Alternative splicing of *CD40* in various animals.
**Fig. S14** Alternative splicing and alternative transcription start sites of various animal *NOSTRIN* genes.
**Table S1** Animal species included in the reduced web‐mode database of alternative isoforms.
**Table S2** Conserved *Arabidopsis thaliana* alternative splicing events used as an initial source for the training set for the machine learning algorithm.
**Table S3** AGI codes and accession numbers of validated plant alternative proteins.
**Table S4** The full list of analyzed isoform pairs from animals, in the order corresponding to the graph presented in Fig. 3(b).
Please note: Wiley is not responsible for the content or functionality of any Supporting Information supplied by the authors. Any queries (other than missing material) should be directed to the *New Phytologist* Central Office.

## Data Availability

The code of the algorithm is available at GitHub (https://github.com/kdcd/catsnap).
