# Supplementary material for: Catsnap: a user‐friendly algorithm for determining the conservation of protein variants reveals extensive parallelisms in the evolution of alternative splicing
Source: New Phytol. 2023 Feb 28;238(4):1722–32. doi: 10.1111/nph.18799 (PMC10952736; doi:10.1111/nph.18799)
Supplement: Supplementary file 1 — Fig. S1 The outline of the Catsnap machine learning features. Fig. S2 The snapshots of the Catnap graphical output interface. Fig. S3 Schematic relationships of the main plant and animal phylogenetic groups. Fig. S4 Alternative splicing of TTL from representative plant species. Fig. S5 Alternative splicing of RCA in various plants. Fig. S6 Alternative splicing of JAZ10 in various plants. Fig. S7 Alternative splicing and alternative transcription start sites of SGR5 in various plants. Fig. S8 Alternative splicing of CPK28 in various plants. Fig. S9 Alternative splicing of PTB2 in various plants. Fig. S10 Alternative splicing of TFIIIA in various plants. Fig. S11 Alternative splicing of Glu4 in various animals. Fig. S12 Alternative splicing of Kif2a in various animals. Fig. S13 Alternative splicing of CD40 in various animals. Fig. S14 Alternative splicing and alternative transcription start sites of various animal NOSTRIN genes. Table S1 Animal species included in the reduced web‐mode database of alternative isoforms. Table S2 Conserved Arabidopsis thaliana alternative splicing events used as an initial source for the training set for the machine learning algorithm. Table S3 AGI codes and accession numbers of validated plant alternative proteins. Table S4 The full list of analyzed isoform pairs from animals, in the order corresponding to the graph presented in Fig. 3(b). Please note: Wiley is not responsible for the content or functionality of any Supporting Information supplied by the authors. Any queries (other than missing material) should be directed to the New Phytologist Central Office. [file NPH-238-1722-s001.pdf]

***New Phytologist* Supporting Information**

Article title: Catsnap: a user-friendly algorithm for determining the conservation of protein variantsreveals extensive parallelisms in the evolution of alternative splicing

Authors: Ksenia Timofeyenko, Dzmitry Kanavalau, Panagiotis Alexiou, Maria Kalyna, and Kamil
Růžička

Article acceptance date: January 27, 2023

The following Supporting information is available for this article:

Fig. S1 The outline of the Catsnap ML features.

Fig. S2 The snapshots of the Catnap graphical output interface.

Fig. S3 Schematic relationships of the main plant (a) and animal (b) phylogenetic groups.

Fig. S4 Alternative splicing of *TTL* from representative plant species.

Fig. S5 Alternative splicing of *RCA* in various plants.

Fig. S6 Alternative splicing of *JAZ10* in various plants.

Fig. S7 Alternative splicing and alternative transcription start sites of *SGR5* in various plants.

Fig. S8 Alternative splicing of *CPK28* in various plants.

Fig. S9 Alternative splicing of *PTB2* in various plants.

Fig. S10 Alternative splicing of *TFIIIA* in various plants.

Fig. S11 Alternative splicing of *Glu4* in various animals.

Fig. S12 Alternative splicing of *Kif2a* in various animals.

Fig. S13 Alternative splicing of *CD40* in various animals.

Fig. S14 Alternative splicing and alternative transcription start sites of various animal *NOSTRIN* genes.

Table S1 Animal species included in the reduced web-mode database of alternative isoforms.

Table S2 Conserved *Arabidopsis thaliana* AS events used as an initial source for the training set for the ML algorithm.

Table S3 AGI codes and accession numbers of validated plant alternative proteins.

Table S4 The full list of analyzed isoform pairs from animals, in the order corresponding to the graph presented in Fig. 3b.

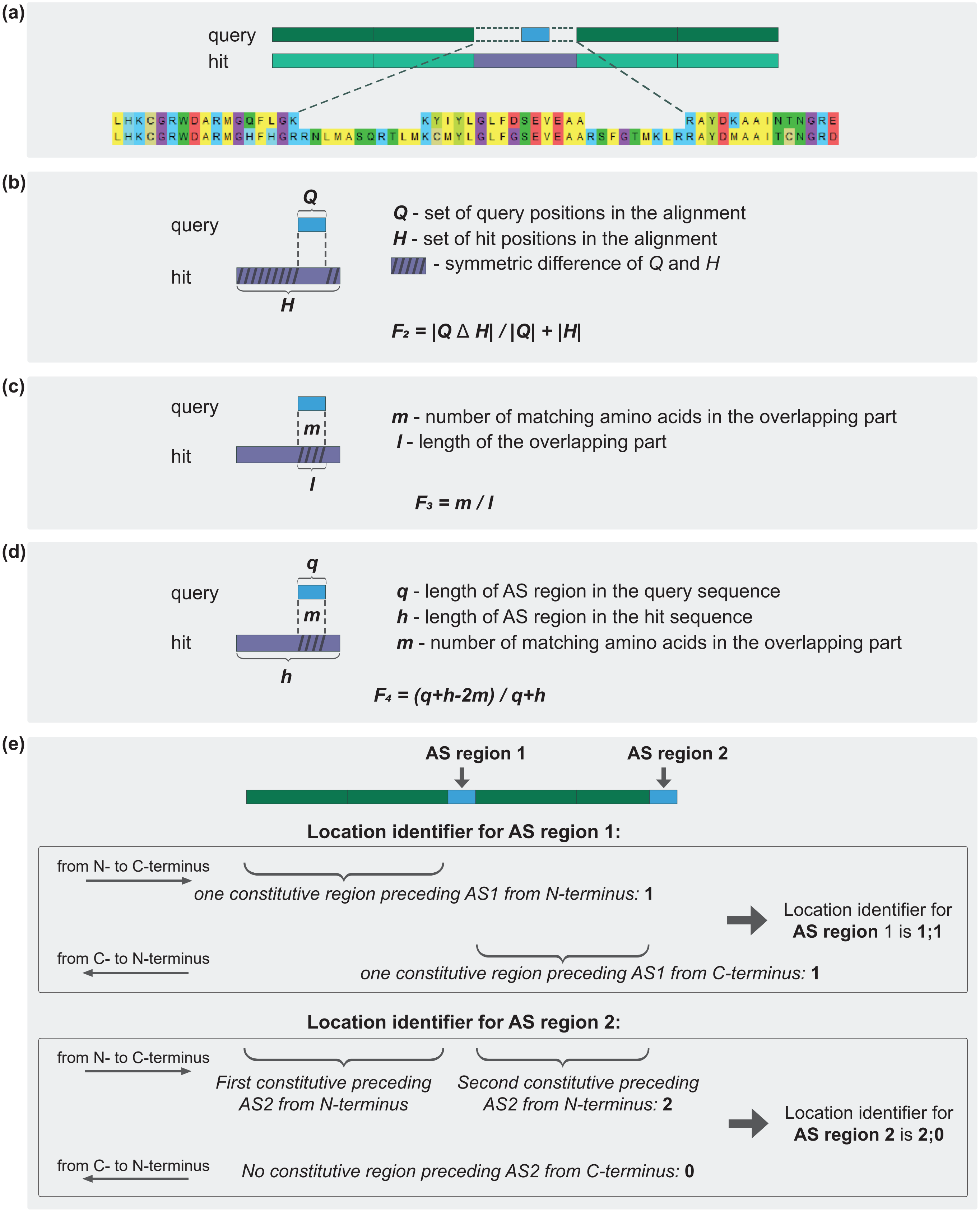

Fig. S1 The outline of the Catsnap ML features. (a) An example AS event illustrating the features implemented by Catsnap. (b) A scheme of the feature emphasizing mutual exclusivity of AS regions between query and hit sequences. (c) and (d) A scheme of the feature weighing amino acid similarity of the AS region (c) and amino acid dissimilarity of the AS regions (d). (e) A diagram describing location identifier used for the analysis of complex AS events.

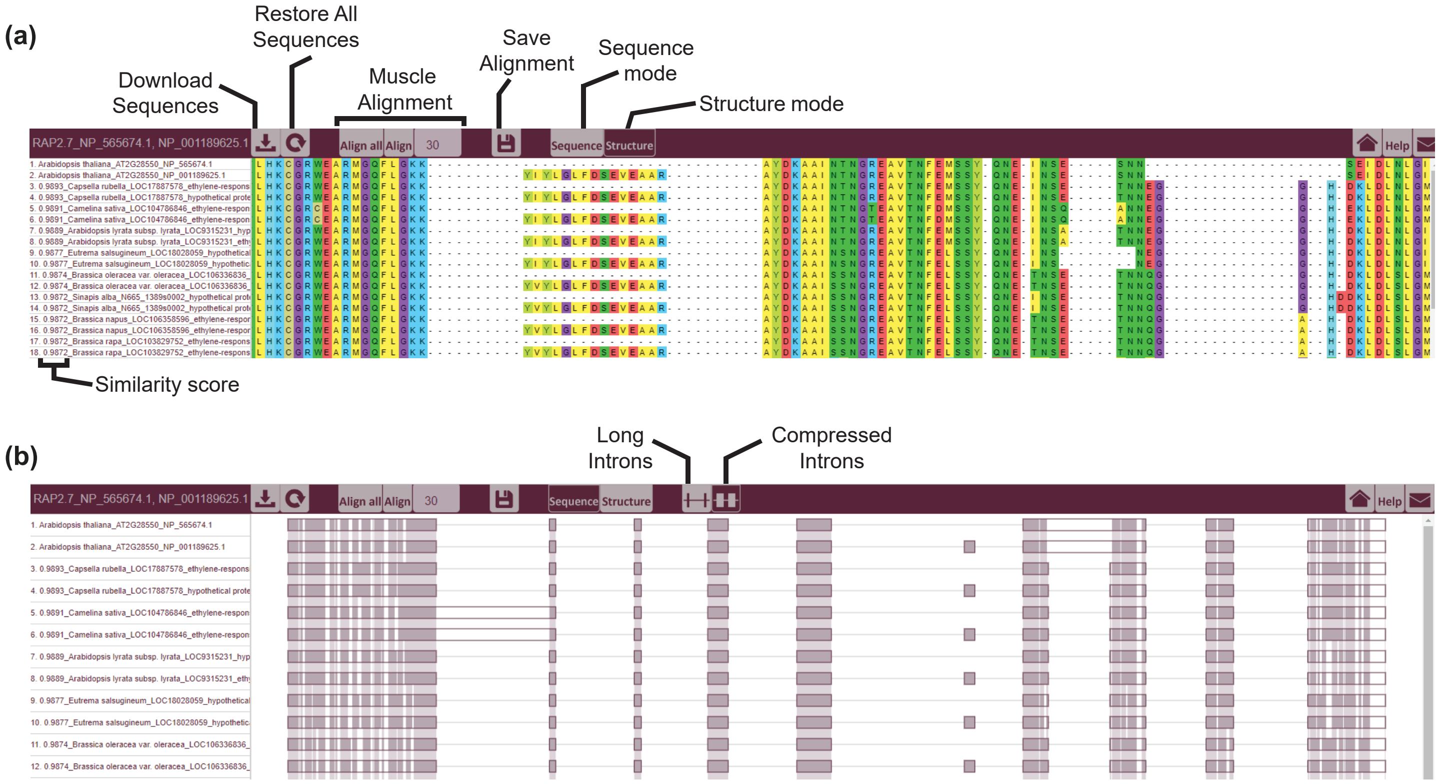

Fig. S2 The snapshots of the Catnap graphical output interface. (a) In the 'Sequence' mode, the candidate orthologous pairs can be downloaded in the FASTA format by choosing 'Download Sequences' from the menu. The .zip archive contains two data sets: all orthologous pairs of isoforms and the reduced list with each most similar isoform per species. The isoforms (the reduced data set) can also be aligned directly by the web browser using the icons 'Muscle Alignment'. The obtained multiple sequence alignment can be elementarily edited (sequences can be deleted, renamed, or moved), dismissed by ‘Restore All Sequences’ or saved by the 'Save Alignment' icon. For the additional instructions, refer to the 'Help' button. (b) The snapshot of the ‘Structure’ mode shows exon-intron schemes of the identified isoforms. They are drawn on the basis of the alignment from the ‘Sequence’ mode. Exons are indicated as rectangles, introns as lines. White regions inside exons correspond to the gaps in the protein alignment (may thereby affect the true proportionality of the resulting schemes); in the instances when multiple exons are merged, they are joined by a solid frame. The buttons denoting 'Long Introns' and 'Compressed Introns' switch between the representations of the diagrams, allowing for a convenient viewing of the AS events particularly in organisms with long introns (typically animals).

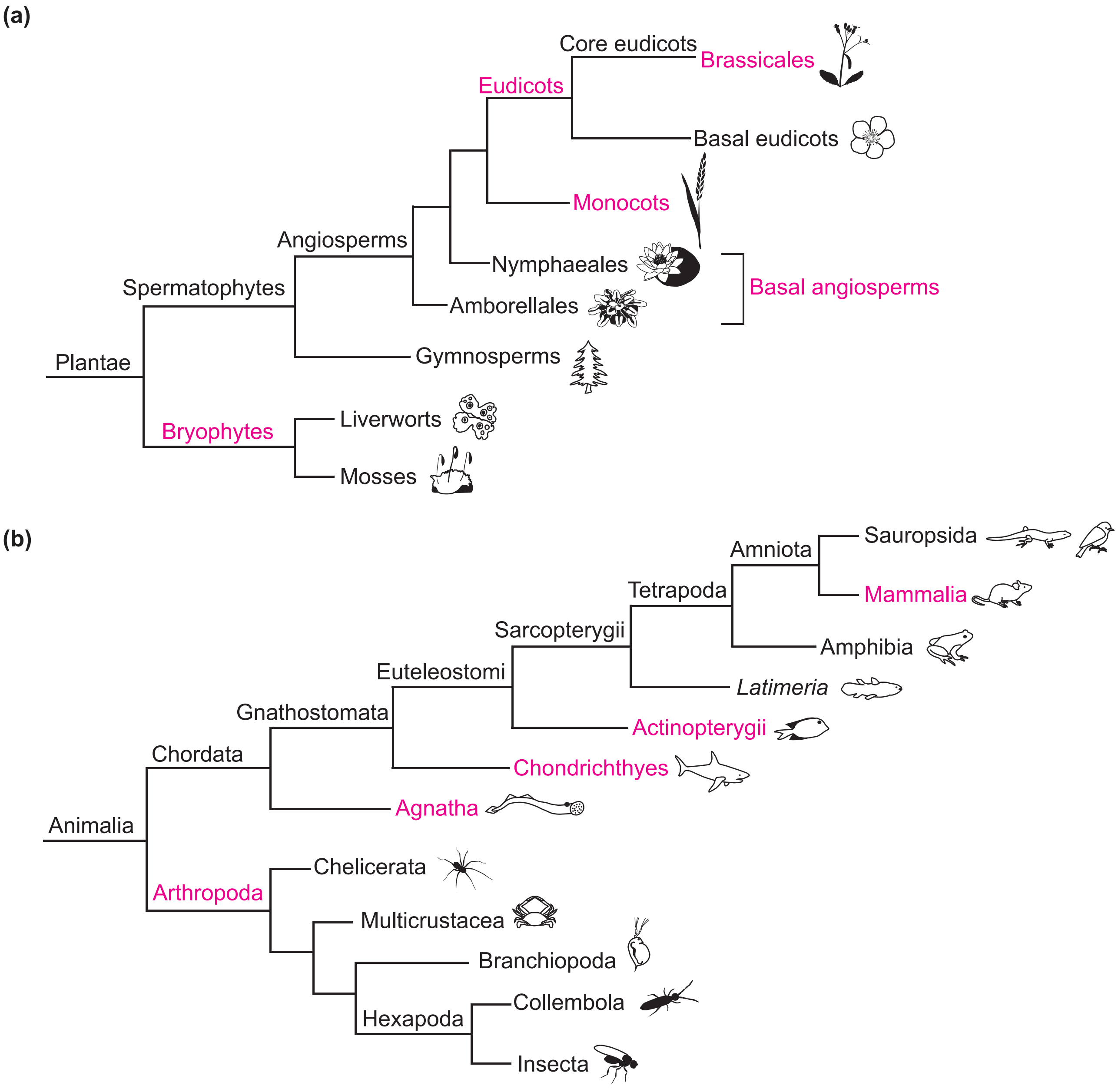

Fig. S3 Schematic relationships of the main plant (a) and animal (b) phylogenetic groups. The group names used in the columns 'Evolutionary depth' in the main figures are indicated in pink.

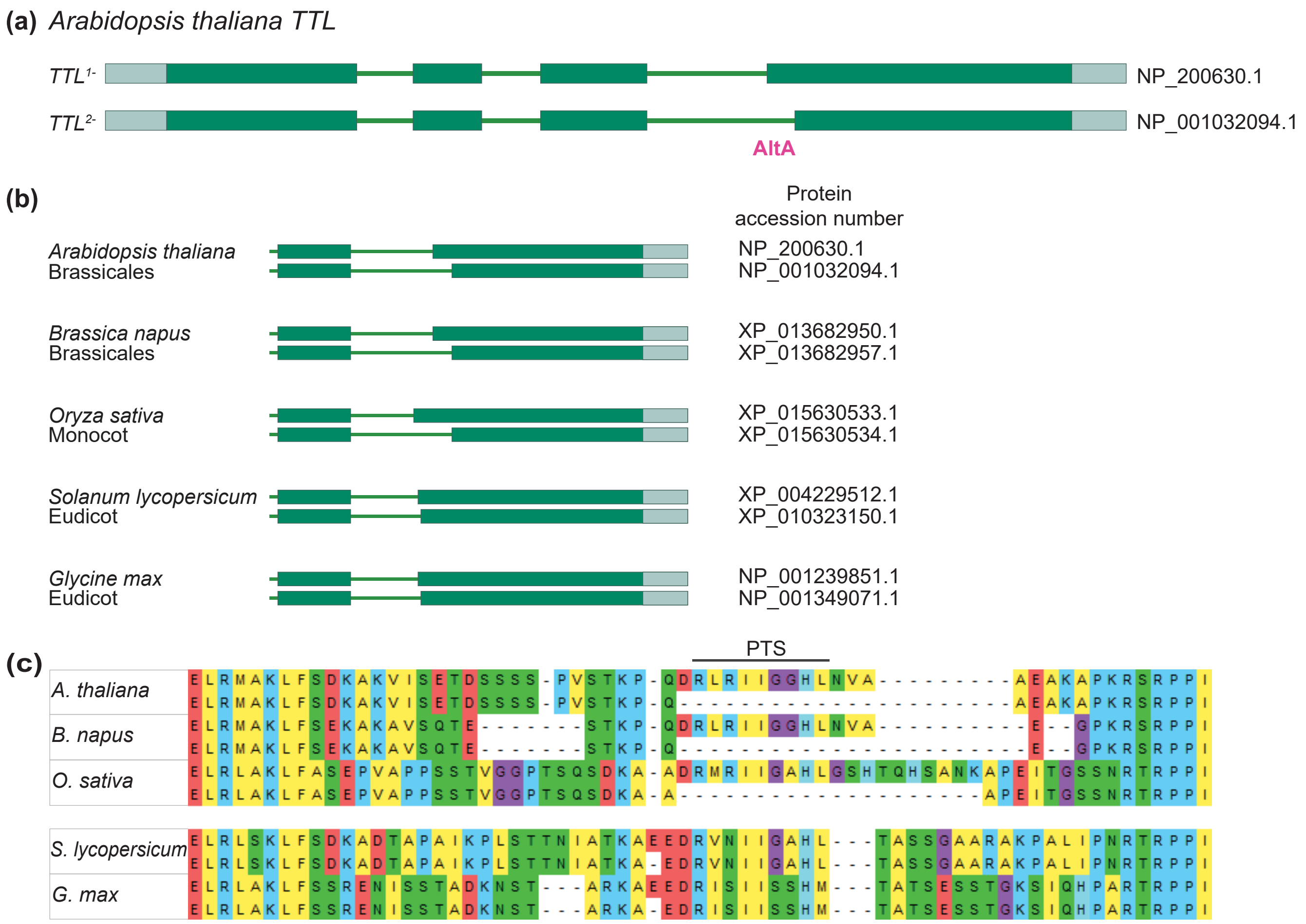

Fig. S4 Alternative splicing of *TTL* from representative plant species. (a) An exon-intron scheme of the *TTL*$^{1-}$ and *TTL*$^{2-}$ splice variants from *Arabidopsis thaliana*. (b) Multiple species show AltA in the corresponding intron. (c) Amino acid sequence alignment of the region undergoing AS from selected species. Depending on the organism, AltA introduces either the peroxisome targeting signal (PTS) or a single glutamate residue (E).

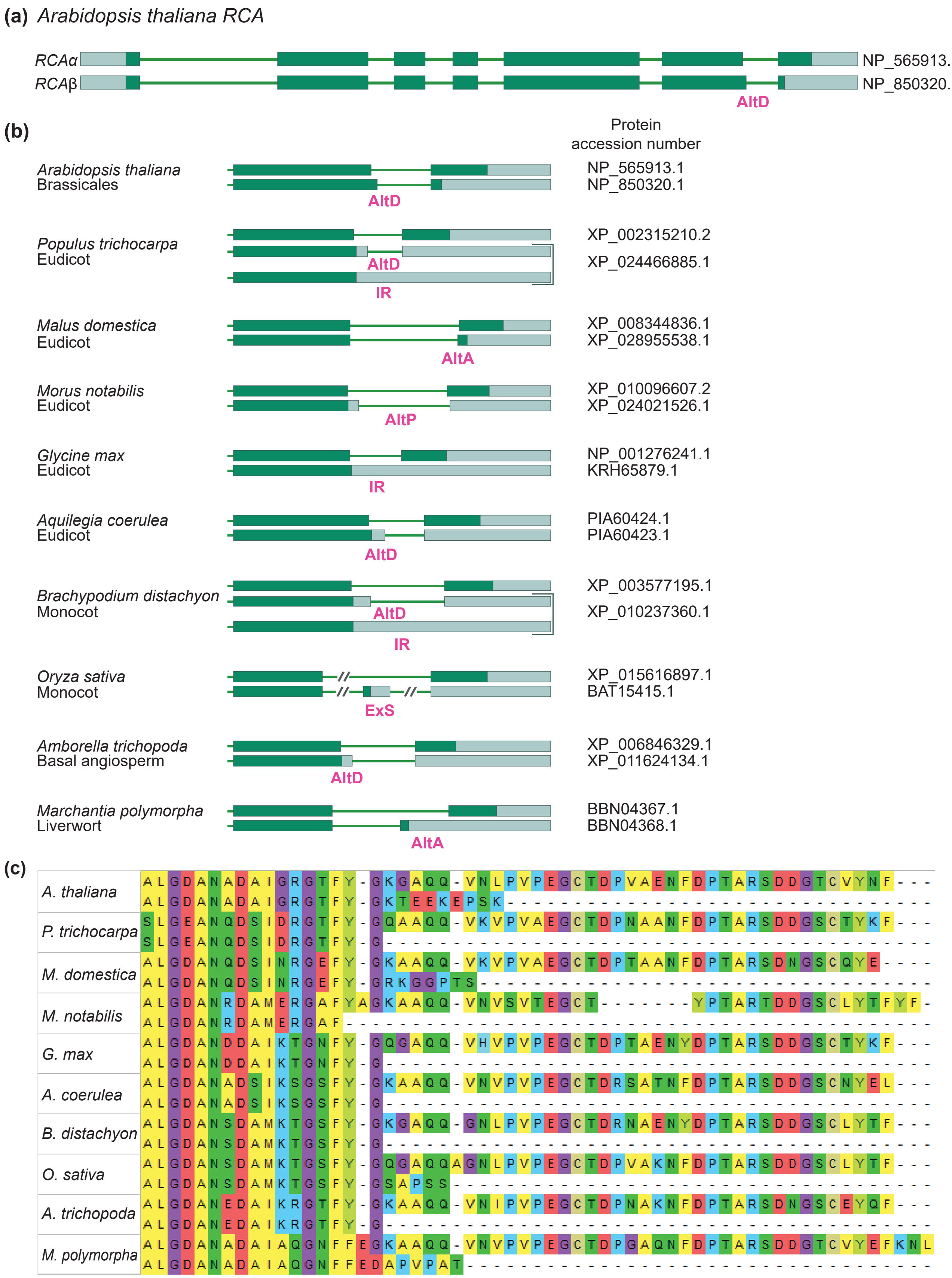

Fig. S5 Alternative splicing of *RCA* in various plants. (a) A scheme of *RCAα* and *RCAβ* splice variants from *Arabidopsis thaliana*. (b) Conservation of the truncated RCAβ isoforms produced by various AS types, depending on the species, as returned by Catsnap. (c) Amino acid sequence alignment of the C-terminal parts of the RCAα and RCAβ proteins.

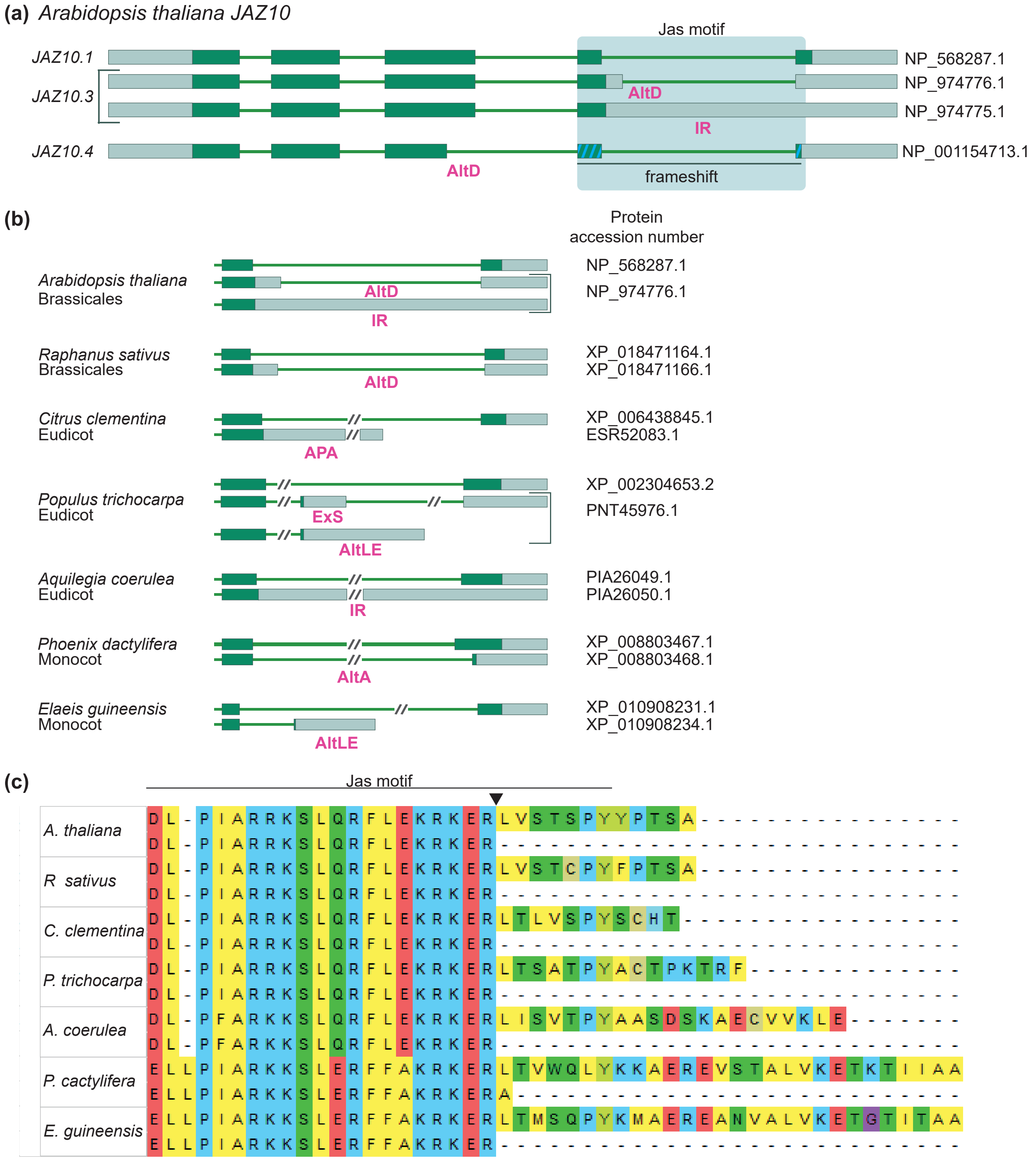

Fig. S6 Alternative splicing of *JAZ10* in various plants. (a) A scheme of the *JAZ10* gene producing canonical JAZ10.1, and alternative JAZ10.3 (encoded by two transcripts) and JAZ10.4 isoforms. AS affects the Jas motif at the C-terminus (boxed in green), required for protein-protein interactions crucial for jasmonate signaling. The Jas motif of JAZ10.3 is partially truncated due to a premature stop codon introduced by either AltD or IR. In JAZ10.4, the whole Jas motif is replaced by a frameshifted sequence, introduced by AltD in the third intron. (b) Conserved truncation of the Jas motif attributable to the JAZ10.3 isoform in the last intron results from various AS types in different species. (c) Alignment of selected C-terminal parts of the JAZ10.1 and JAZ10.3 amino acid sequences from various species. Arrowhead indicates the position of the respective exon junction.

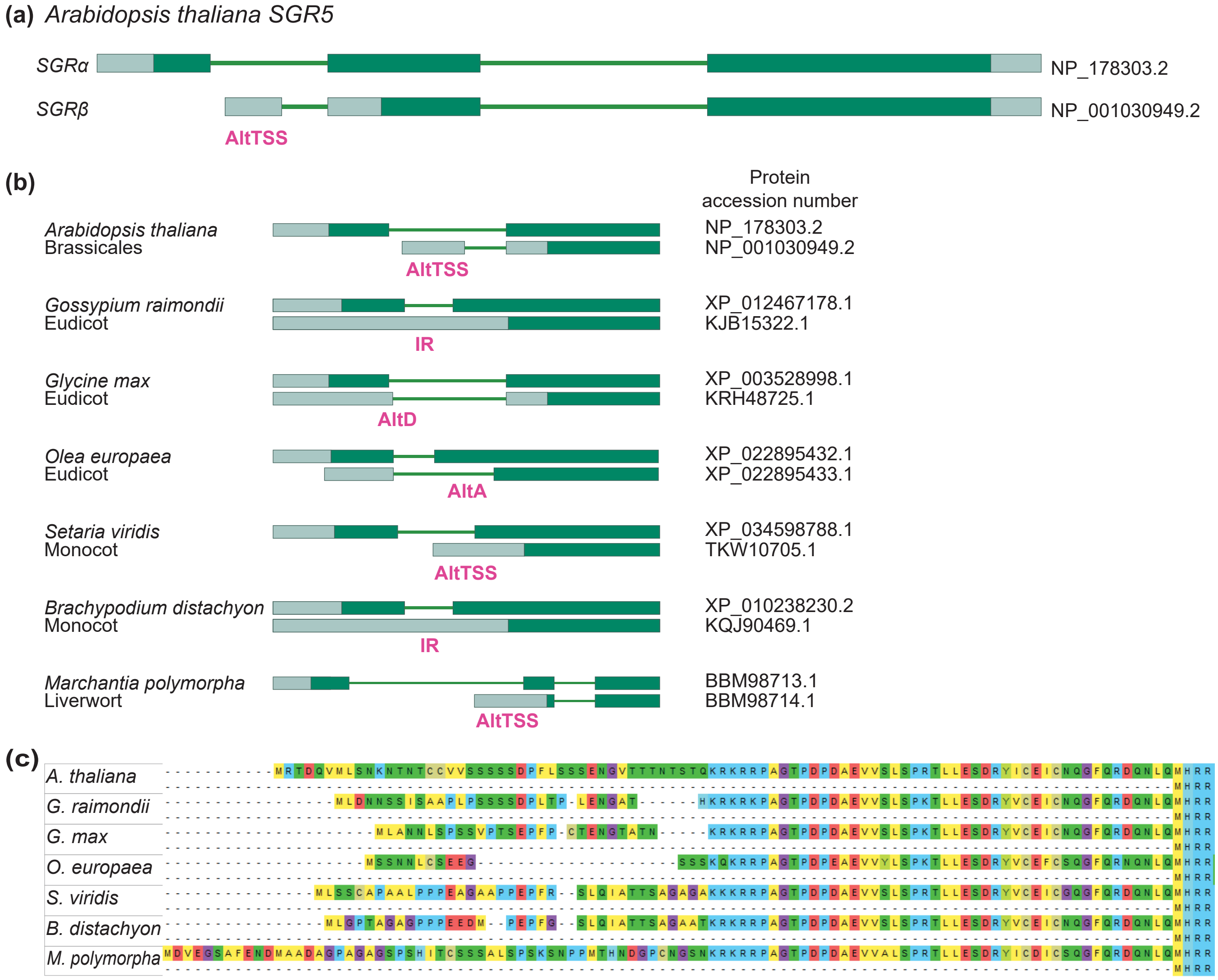

Fig. S7 Alternative splicing and alternative transcription start sites of *SGR5* in various plants. (a) A scheme of the *SGR5 α* and *SGR5β* transcript variants from *Arabidopsis thaliana*. (b) The N-terminal truncation of SGR5β in different species originates from various AS types and alternative transcription start sites. (c) Amino acid sequence alignment of the N-terminal part of SGR5 from the species listed on (b).

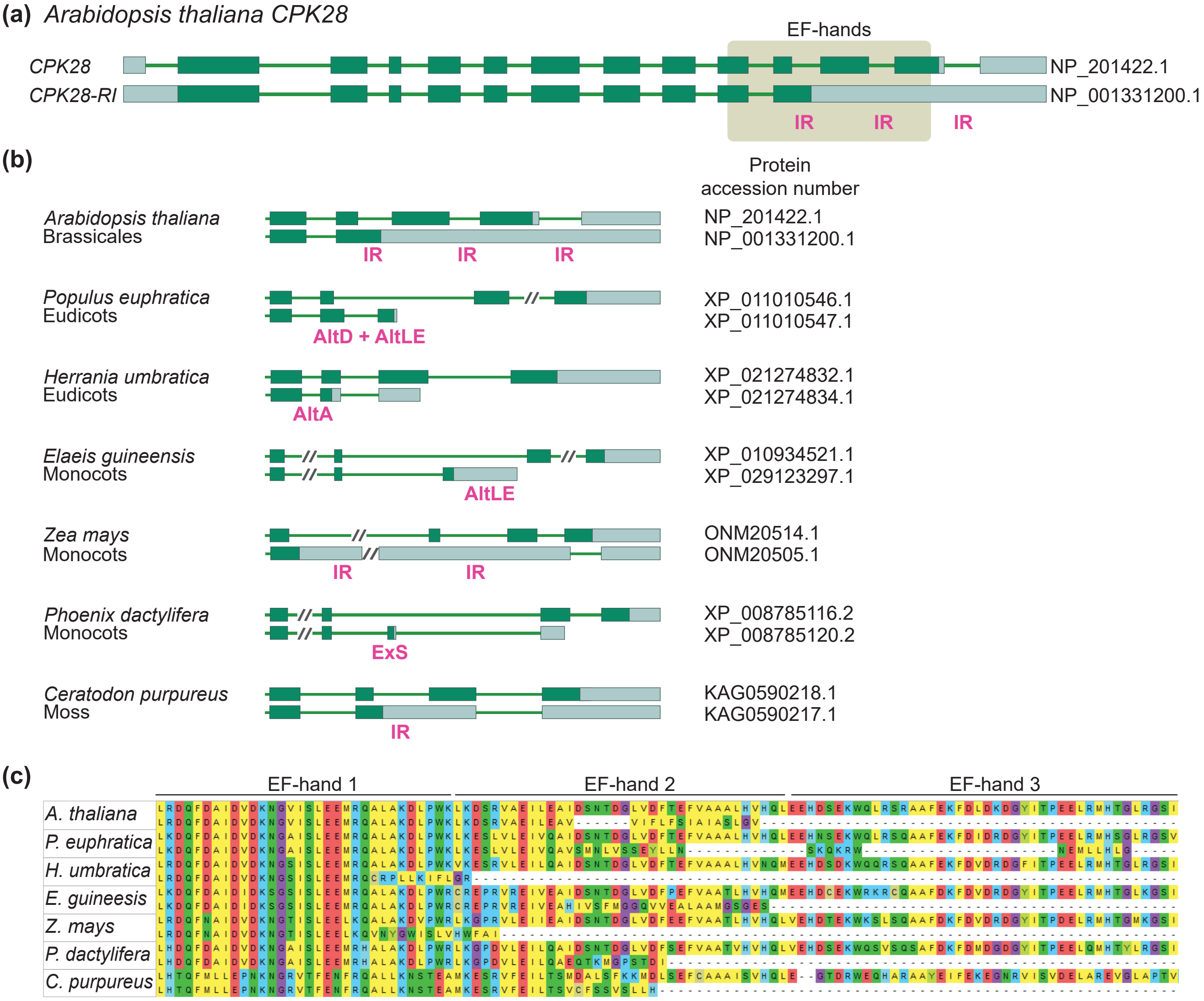

Fig. S8 Alternative splicing of *CPK28* in various plants. (a) A scheme of a triple IR event in the *Arabidopsis thaliana CPK28*. (b) Conserved isoforms lacking the encoded EF-hands motifs result from various AS types. (c) Amino acid alignment of selected CPK28 and CPK28-RI isoforms. Three out of a total of four EF-hand domains responsible for the Ca^{2+}-dependent activation in the area of the C-terminus of the CPK28 kinase are marked on the top of the alignment.

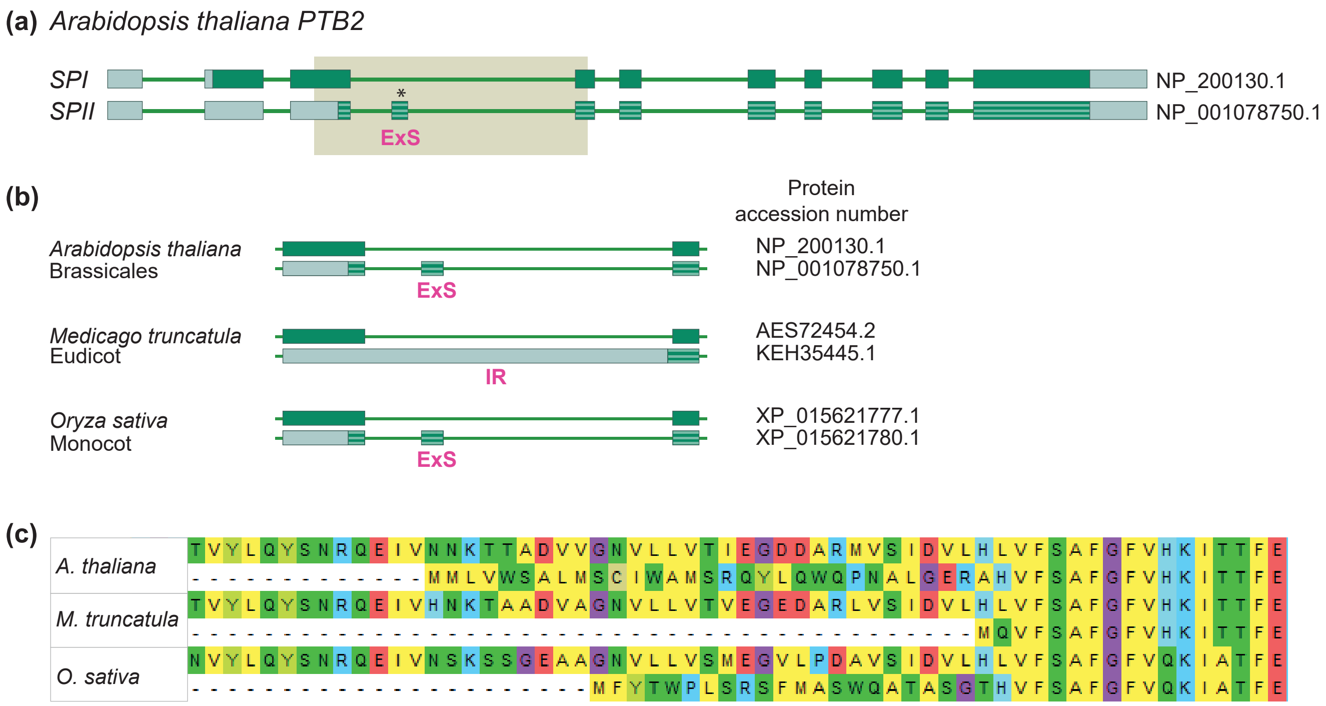

Fig. S9 Alternative splicing of *PTB2* in various plants. (a) A scheme of the exon skipping event in the *Arabidopsis thaliana PTB2*. (b) The transcripts that include the premature termination codon (PTC) are conserved in eudicots and monocots and can involve various AS types. (c) A sequence alignment of the nominal proteins, whose transcripts are subjected to NMD. The light green rectangle on (a) corresponds to the area of the alignment on (c). The asterisk marks PTC referring to the frame of the reference isoform. The nominal open reading frame in alternative isoforms is determined as the longest one in the sequence context (horizontal hatching), as defined by RefSeq.

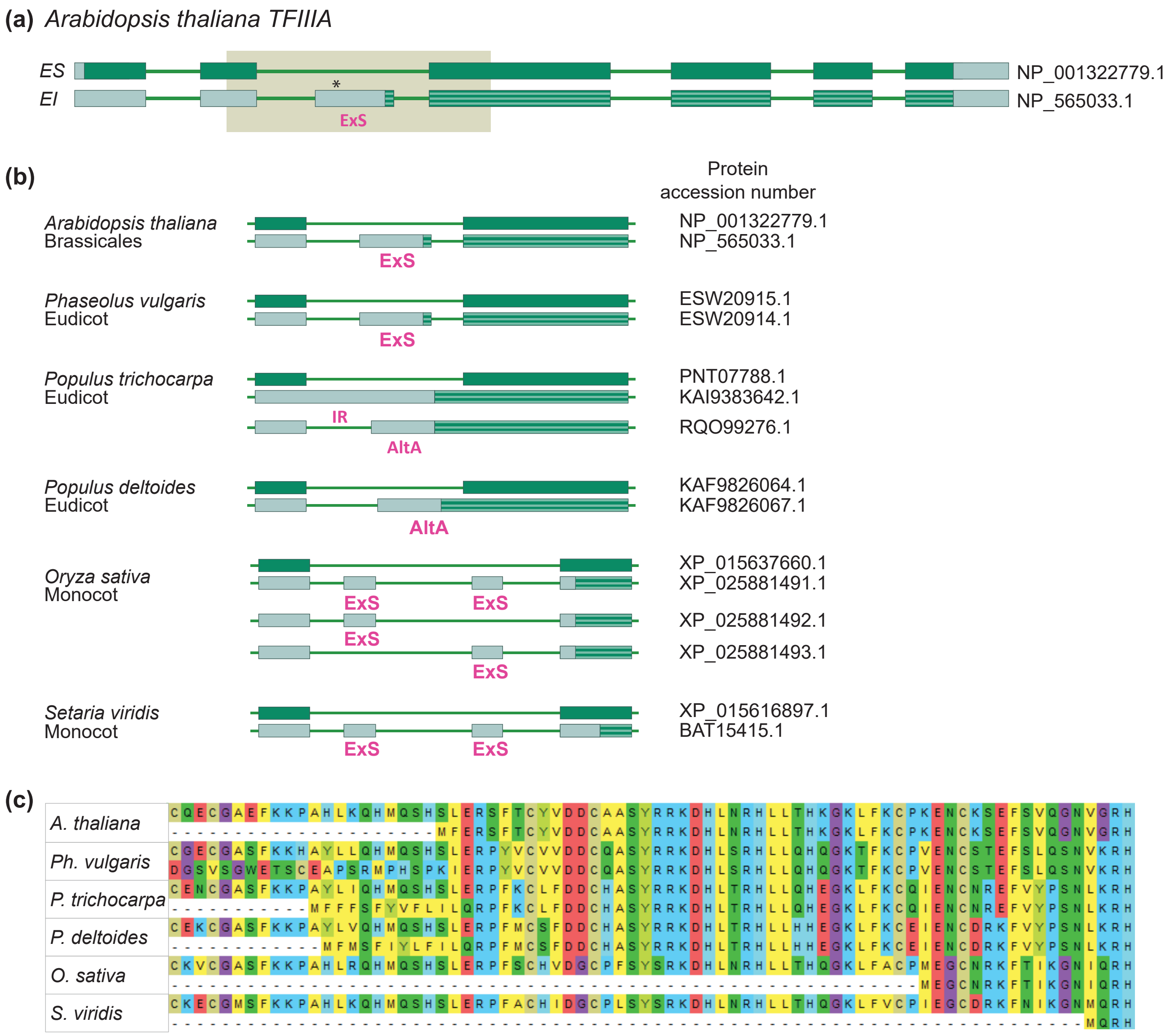

Fig. S10 Alternative splicing of *TFIIIA* in various plants. (a) An exon-intron scheme of the exon skipping (*ES*) and the exon including (*EI*) transcripts of the *TFIIIA* gene in *Arabidopsis thaliana*. (b) The NMD-triggering PTC originates from various AS types in different species. (c) A sequence alignment of the predicted amino acid sequences, shortened by the PTC. The light green rectangle on (a) corresponds to the area of the alignment on (c). The asterisk marks PTC. The open reading frame is determined as the longest one in the sequence context (horizontal hatching), as defined by RefSeq.

(a) *Rattus norvegicus GluR4*

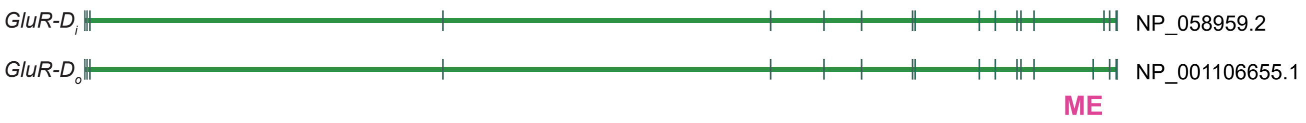

(b)

Group	Species	Protein accesion number
Mammalia	*Rattus norvegicus*	NP_058959.2, NP_001106655.1
Aves	*Gallus gallus*	NP_001106657.1, NP_990545.1
Reptilia	*Crotalus tigris*	XP_039177701.1, XP_039177635.1
Amphibia	*Xenopus laevis*	XP_041440492.1, XP_041440491.1
Actinopterygii	*Salmo salar*	XP_045580724.1, XP_045580725.1
Chondrichthyes	*Scyliorhinus canicula*	XP_038673817.1, XP_038673816.1

(c)

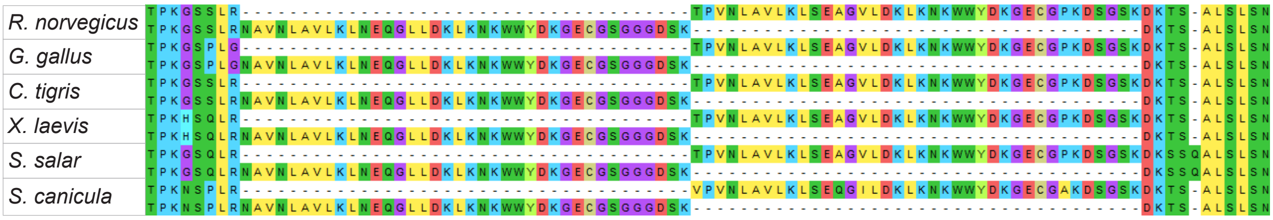

Fig. S11 Alternative splicing of *Glu4* in various animals. (a) A scheme of *GluR-D$_i$* and *GluR-D$_o$* splice variants differing by the mutually exclusive exons (ME) from rat (*Rattus norvegicus*). (b) Accession numbers of conserved isoform pairs from selected species returned by Catsnap. (c) Amino acid sequence alignment of the C-terminal region of GluR-D$_i$ and GluR-D$_o$ from the proteins listed on (b).

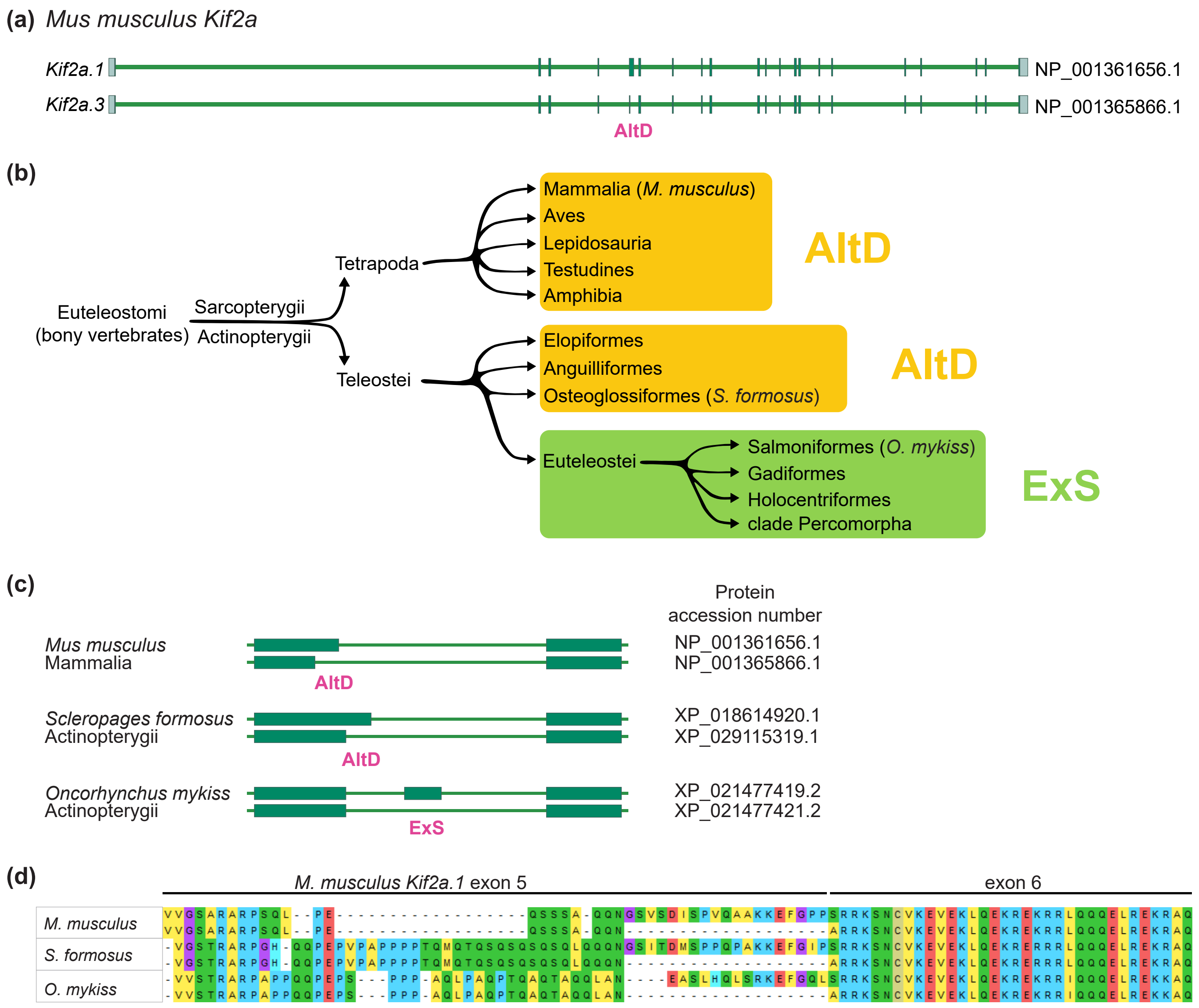

Fig. S12 Alternative splicing of *Kif2a* in various animals. (a) A scheme the mouse (*Mus musculus*) canonical *Kif2a.1* and alternative splice variant *Kif2a.3*, produced by AltD. (b) Evolutionary cladogram depicting AS of *Kif2a* in various animal phylogenetic groups. Protein isoforms corresponding to mouse Kif2A.3 are processed by AltD in tetrapods and basal teleosts, however, evolutionarily derived teleosts display ExS in this gene. (c) Exon-intron diagrams of representative Kif2a transcripts from tetrapods (*Mus musculus*), basal teleosts (*Scleropages formosus*), and euteleosts (*Oncorhynchus mykiss*). (d) Amino acid sequence alignment of the region of Kif2A processed by AS in the species presented in (c).

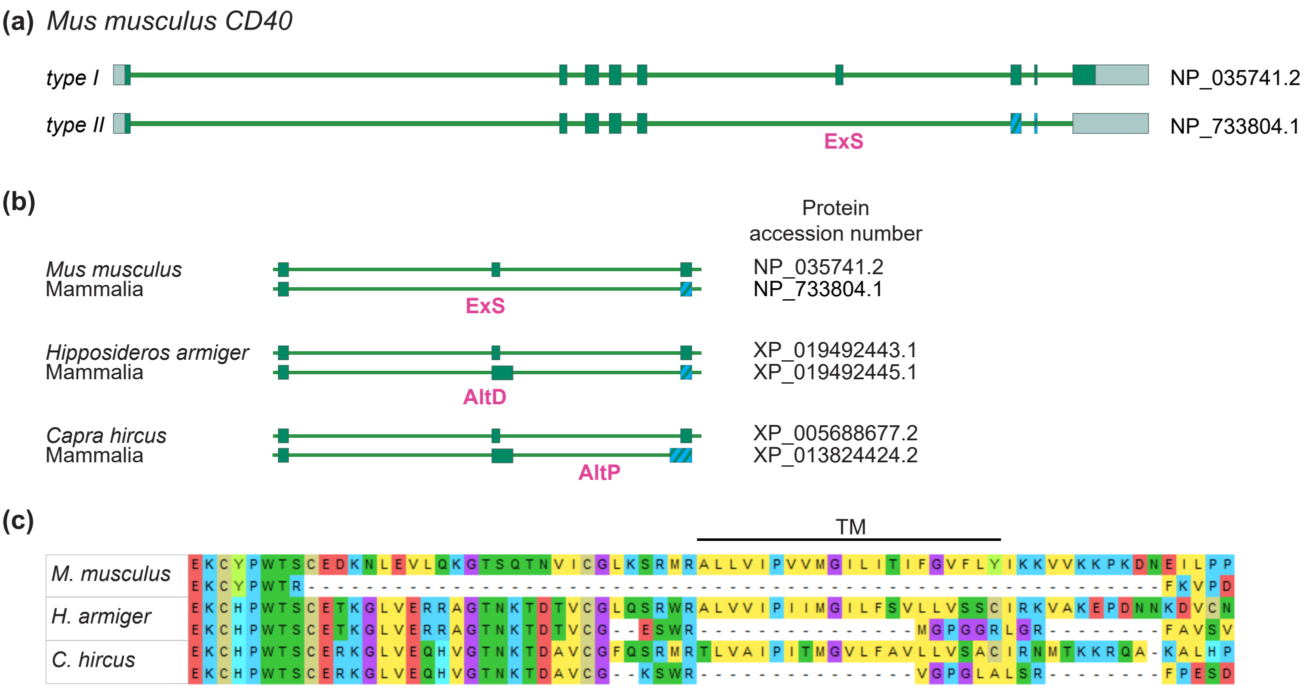

Fig. S13 Alternative splicing of *CD40* in various animals. (a) A scheme of the mouse (*Mus musculus*) *CD40 type I* and *type II* splice variants. (b) The transmembrane domain (TM) is removed by a frame shift (hatched) resulting from various AS types in mammals. (c) Amino acid sequence alignment of the region undergoing AS from selected species.

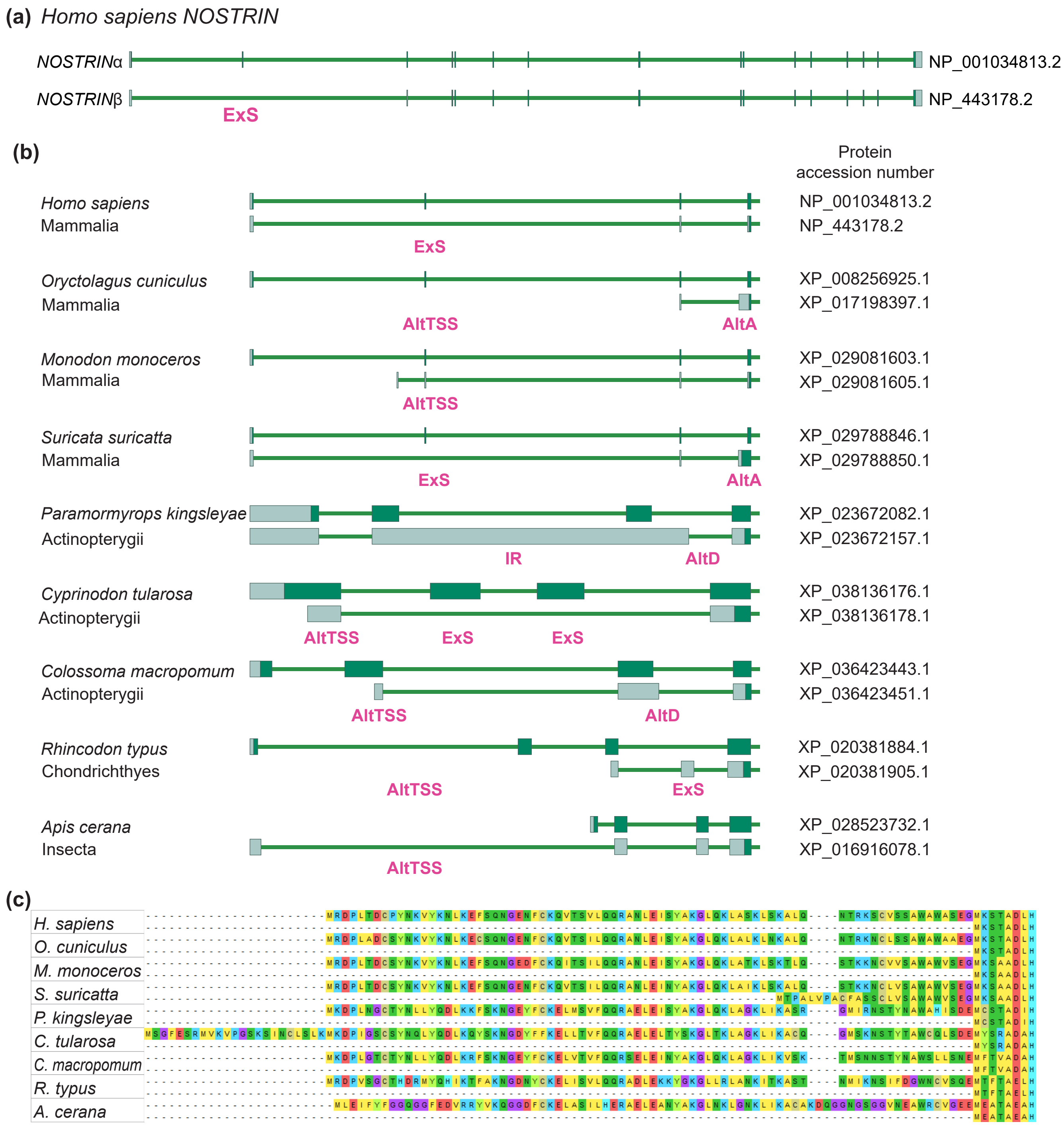

Fig. S14 Alternative splicing and alternative transcription start sites of various animal *NOSTRIN* genes. (a) A scheme of *NOSTRINα* and *NOSTRINβ* from human (*Homo sapiens*). (b) The N-terminal truncation in NOSTRIN is controlled by various AS types, including their combinations and AltTSS. (c) Amino acid sequence alignment of the N-terminal parts of NOSTRIN from the genes outlined in (b).

Table S1 Animal species included in the reduced web-mode database of alternative isoforms.

Group	Species name		
Mammalia	*Homo sapiens*<br>*Macaca mulatta*<br>*Mus musculus*<br>*Rattus norvegicus*<br>*Ovis aries*<br>*Balaenoptera musculus*<br>*Bos taurus*<br>*Orcinus orca*	*Equus caballus*<br>*Felis catus*<br>*Zalophus californianus*<br>*Myotis myotis*<br>*Pteropus giganteus*<br>*Choloepus didactylus*<br>*Manis pentadactyla*	*Loxodonta africana*<br>*Trichechus manatus latirostris*<br>*Erinaceus europaeus*<br>*Orycteropus afer afer*<br>*Monodelphis domestica*<br>*Ornithorhynchus anatinus*<br>*Tachyglossus aculeatus*
Aves	*Gallus gallus*<br>*Falco rusticolus*<br>*Tyto alba*<br>*Egretta garzetta*	*Passer montanus*<br>*Columba livia*<br>*Calidris pugnax*<br>*Melopsittacus undulatus*	*Aquila chrysaetos chrysaetos*<br>*Aptenodytes forsteri*<br>*Picoides pubescens*<br>*Struthio camelus australis*
Reptilia	*Chelonia mydas*<br>*Mauremys reevesii*	*Crotalus tigris*<br>*Podarcis muralis*	*Alligator mississippiensis*
Amphibia	*Xenopus laevis*	*Rana temporaria*	
Actinopterygii	*Protopterus annectens*<br>*Latimeria chalumnae*<br>*Polypterus senegalus*<br>*Acipenser ruthenus*<br>*Anguilla anguilla*<br>*Scleropages formosus*<br>*Carassius auratus*	*Danio rerio*<br>*Pygocentrus nattereri*<br>*Oncorhynchus mykiss*<br>*Salmo salar*<br>*Gadus morhua*<br>*Myripristis murdjan*	*Siniperca chuatsi*<br>*Sander lucioperca*<br>*Oreochromis niloticus*<br>*Hippoglossus hippoglossus*<br>*Thunnus albacares*<br>*Hippocampus comes*
Chondrichthyes	*Scyliorhinus canicula*	*Chiloscyllium plagiosum*	
Agnatha	*Petromyzon marinus*		
Cephalochordata	*Branchiostoma belcheri*	*Branchiostoma floridae*	
Ascidiacea	*Styela clava*	*Ciona intestinalis*	
Echinodermata	*Acanthaster planci*		
Crustacea	*Procambarus clarkii*	*Daphnia magna*	
Chelicerata	*Varroa destructor*	*Limulus polyphemus*	*Parasteatoda tepidariorum*
Insecta	*Bombyx mori*<br>*Photinus pyralis*<br>*Thrips palmi*	*Nilaparvata lugens*<br>*Apis mellifera*<br>*Folsomia candida*	*Chrysoperla carnea*<br>*Zootermopsis nevadensis*<br>*Drosophila melanogaster*
Mollusca	*Crassostrea gigas*	*Pomacea canaliculata*	*Octopus vulgaris*
Cnidaria	*Acropora millepora*	*Hydra vulgaris*	*Dendronephthya gigantea*
Nematoda	*Caenorhabditis elegans*		
Platyhelminthes	*Schistosoma mansoni*		
Scalidophora	*Priapulus caudatus*		
Porifera	*Amphimedon queenslandica*		
Placozoa	*Trichoplax adhaerens*		
Unicellular	*Sphaeroforma arctica JP610*<br>*Fonticula alba*	*Capsaspora owczarzaki ATCC 30864*<br>*Salpingoeca rosetta*	

Table S2 Conserved *Arabidopsis thaliana* AS events used as an initial source for the training set for the ML algorithm.

**Locus tag**	**Protein pair accession number**
AT4G25500	NP_001078447.1, NP_194280.1
AT4G24740	NP_001329102.1, NP_194205.1
AT4G24740	NP_001329105.1, NP_194205.1
AT2G28550	NP_565674.1, NP_001189625.1
AT1G17720	NP_564033.1, NP_849681.1
AT3G29390	NP_566850.3, NP_001326115.1
AT5G58220	NP_001032094.1, NP_200630.1
AT1G23080	NP_849700.1, NP_001077584.1
AT1G55870	NP_001322686.1, NP_175983.5
AT3G27110	NP_001325723.1, NP_566808.1
AT5G06440	NP_196262.3, NP_001332440.1
AT5G61540	NP_974974.1, NP_200962.2
AT4G36690	NP_195387.1, NP_849509.1
AT2G44480	NP_001118525.1, NP_001324585.1
AT1G02090	NP_563645.1, NP_849576.1
AT3G01150	NP_186764.1, NP_001319439.1
AT3G01150	NP_186764.1, NP_001327637.1
AT3G06550	NP_001118592.1, NP_187307.3
AT2G39730	NP_565913.1, NP_850320.1
AT3G46130	NP_190199.2, NP_001030816.1
AT3G46130	NP_190199.2, NP_001078249.1
AT2G46830	NP_850460.1, NP_001318437.1
AT5G63120	NP_001331871.1, NP_974985.1
AT2G20585	NP_001324661.1, NP_001324662.1
AT4G16695	NP_849396.1, NP_001328627.1
AT4G17310	NP_001329195.1, NP_001329196.1
AT5G57630	NP_001330673.1, NP_001330674.1
AT5G57630	NP_001330673.1, NP_568860.1
AT5G10630	NP_001331941.1, NP_001331943.1
AT5G10630	NP_001331941.1, NP_196625.2
AT5G10630	NP_001331941.1, NP_001331941.1

Table S3 AGI codes and accession numbers of validated plant alternative proteins.

Isoform 1	Isoform 2	AGI code	RefSeq protein accession numbers
RCAα	RCAβ	AT2G39730.1, AT2G39730.2	NP_565913.1, NP_850320.1
JAZ10.1	JAZ10.3	AT5G13220.1, AT5G13220.3	NP_568287.1, NP_974776.1
JAZ10.1	JAZ10.4	AT5G13220.1, AT5G13220.4	NP_568287.1, NP_001154713.1
SGR5α	SGR5β	AT2G01940.3, AT2G01940.2	NP_178303.2, custom
IDD14α	IDD14β	AT1G68130.1, AT1G68130.2	NP_176980.1, NP_001077791.1
CCA1α	CCA1β	AT2G46830.1, AT2G46830.2	NP_850460.1, custom
BES1-L	BES1-S	AT1G19350.1, AT1G19350.3	NP_564081.1, NP_973863.1
FLM-β	FLM-δ	AT1G77080.4, AT1G77080.2	NP_177833.3, NP_850979.1
MAF2var1	MAF2var5	AT5G65050.3, AT5G65050.2	NP_001119498.1, NP_001078798.1
SEP3.2	SEP3.3	AT1G24260.2, AT1G24260.3	NP_564214.2, NP_001185081.1
ZIFL1.1	ZIFL1.3	AT5G13750.1, AT5G13750.4	NP_851036.1, NP_001031877.1
HAB1.1	HAB2.2	AT1G72770.1, AT1G72770.2	NP_177421.1, NP_001077815.1
ABI2.1	ABI2.2	AT5G57050.1, AT5G57050.3	NP_200515.1, NP_001331640.1
MPK13 Full	MPK13 I4	AT1G07880.2, AT1G07880.1	NP_001030990.1, NP_172266.2
TTL^{1-}	TTL^{2-}	AT5G58220.1, AT5G58220.3	NP_200630.1, NP_001032094.1
YUC4.1	YUC4.2	AT5G11320.1, AT5G11320.2	NP_196693.1, NP_850808.1
SR45.1	SR45.2	AT1G16610.1, AT1G16610.2	NP_173107.1, NP_973844.1
RAD52-1A	RAD52-1B	AT1G71310.2, AT1G71310.3	NP_974123.1, NP_849876.1
XBAT35.1	XBAT35.2	AT3G23280.1, AT3G23280.2	NP_566724.1, NP_850628.1
IBR5.1	IBR5.3	AT2G04550.1, AT2G04550.3	NP_178534.2, NP_973418.2
CPK28	CPK28-RI	AT5G66210.2, AT5G66210.6	NP_201422.1, NP_001331200.1
HsfA2	HsfA2-III	AT2G26150.1, custom	NP_180184.1, custom
ARF8.1	ARF8.4	AT5G37020.1, custom	NP_198518.1, custom
SR45a-1a	SR45a-1b	AT1G07350.1, custom	NP_563787.2, custom
DOG1-α	DOG1-β	AT5G45830.3, AT5G45830.6	NP_199395.2, NP_001330541.1
PIN7a	PIN7b	AT1G23080.3, AT1G23080.1	NP_001319066.1, NP_849700.1
MP	MP11ir	AT1G19850.1, custom	NP_173414.1, custom
ABI3-α	ABI3-β	AT3G24650.1, custom	NP_189108.1, custom
*Os*RLI1b	*Os*RLI1a	Not available	XP_015636273.1, custom
PTB2 SPI	PTB2 SPII	AT5G53180.1, AT5G53180.2	NP_200130.1, NP_001078750.1
TFIIIA ES	TFIIIA EI	AT1G72050.3, AT1G72050.1	NP_001322779.1, NP_565033.1

Table S4 The full list of analyzed isoform pairs from animals, in the order corresponding to the graph presented in Fig. 3b.

N	Isoform 1	Isoform 2	RefSeq protein accession numbers	Species number	Reference
1	GluR-D_i	GluR-D_o	NP_058959.2, NP_001106655.1	348	Mosbacher *et al.* (1994)
2	CRMP E9a	CRMP E9b	NP_730954.2, NP_477307.1	125	Morris *et al.* (2012)
3	Esrp1 2A+CKLP	Esrp1 2A-CKLP	NP_918944.2, XP_006537778.1	258	Yang & Carstens (2017)
4	CMTM8	CMTM8-v2	NP_849199.2, NP_001307237.1	33	Li *et al.* (2007)
5	Nrxn1 4(+)	Nrxn1 4(-)	NP_064648.3, XP_006523879.1	113	Iijima *et al.* (2011)
6	FGF8a	FGF8b	XP_012821935.1, NP_001008163.1	191	Fletcher *et al.* (2006)
7	$TEAD^{F.L.}$	$TEAD^{\Delta E6}$	NP_001160057.2, NP_033372.2	252	Choi *et al.* (2022)
8	TrpA1-C	TrpA1-D	NP_001261601.1, NP_001261600.1	44	Gu *et al.* (2019)
9	WT1 (+KTS)	WT1 (-KTS)	NP_077744.4, NP_077742.3	279	Larsson *et al.* (1995)
10	ANK1 2.1	ANK1 2.2	NP_065209.2, NP_065210.2	94	Davis *et al.* (1992)
11	PXR.1	PXR.2	NP_003880.3, NP_148934.1	24	Lin *et al.* (2009)
12	TMEM16A(ac)	TMEM16A(a)	NP_001365025.1, XP_011543429.1	199	Ko *et al.* (2020)
13	Dyn3aaa	Dyn3baa	NP_612547.1, XP_006250203.1	189	Gray *et al.* (2005)
14	Mef2Dα1	Mef2Dα2	NP_005911.1, XP_006711395.1	173	Sebastian *et al.* (2013)
15	MEF2C γ(+)	MEF2C γ(-)	NP_001180276.1, NP_001351267.1	257	Zhu & Gulick (2004)
16	NMHC-II-B B1 (+)	NMHC-II-B B1 (-)	XP_017170324.1, NP_780469.1	253	Ma *et al.* (2006)
17	NMHC-II-B B2 (+)	NMHC-II-B B2 (-)	XP_017170323.1, NP_780469.1	206	Ma *et al.* (2006)
18	NLG-1	NLG-1ΔB	XP_038957505.1, XP_038957506.1	187	Lee *et al.* (2010)
19	Dyn2 ba	Dyn2 bb	NP_001005360.1, NP_004936.2	230	Liu *et al.* (2008)
20	Kcnma1 STREX	Kcnma1 ZERO	XP_017171361.1, XP_006518656.1	109	McCartney *et al.* (2005)
21	mSin1.1	mSin1.2	NP_001006618.1, NP_077022.1	225	Frias *et al.* (2006)
22	MYH11 5b(+)	MYH11 5b(-)	NP_001035203.1, NP_002465.1	300	Low *et al.* (2006)
23	$Myo1b^a$	$Myo1b^c$	NP_446438.1, XP_038938883.1	297	Laakso *et al.* (2010)
24	Cp2a	Cp2b	NP_001359440.1, NP_001359439.1	293	Kang *et al.* (2005)
25	TFII-Iβ	TFII-IΔ	NP_034495.2, NP_001074217.1	165	Hakre *et al.* (2006)
26	SMRT 37b(+)	SMRT 37b(-)	XP_018090990.1, XP_018091068.1	65	Malartre *et al.* (2006)

27	hnRNP B1	hnRNP A2	NP_001361674.1, NP_058086.2	140	Han *et al.* (2010)
28	ING4_v1	ING4_v2	NP_001121054.1, NP_057246.2	156	Unoki *et al.* (2006)
29	Lpin1α	Lpin1β	NP_766538.2, NP_056578.2	215	Péterfy *et al.* (2005)
30	ErbB4 CYT-1	ErbB4 CYT-2	NP_005226.1, NP_001036064.1	138	Sundvall *et al.* (2007)
31	Noxo1α	Noxo1β	NP_653204.1, NP_751907.1	39	Ueyama *et al.* (2007)
32	KIF1Bβ 6aa(+)	KIF1Bβ 6aa(-)	XP_038965095.1, XP_038965096.1	224	Matsushita *et al.* (2009)
33	mH2A1.1	mH2A1.2	NP_613075.1, NP_613258.2	394	Kustatscher *et al.* (2005)
34	RyR1 ASI(−)	RyR1 ASI(+)	NP_033135.2, XP_006539752.1	108	Tang *et al.* (2015)
35	TAF1-1 (56-59)	TAF1-2	NP_476956.3, NP_996159.1	51	Metcalf & Wassarman (2006)
36	ubKDM1a	ubKDM1a+2a	NP_055828.2, XP_005245843.1	170	Astro *et al.* (2022)
37	ubKDM1a+2a	nKDM1A+2a+8a	XP_005245843.1, NP_001009999.1	71	Astro *et al.* (2022)
38	KIF2A.1	KIF2A.2	NP_001361656.1, NP_001139251.1	367	Akkaya *et al.* (2021)
39	mSMOα	mSMOµ	NP_663508.1, NP_001171304.1	154	Bianchi *et al.* (2005)
40	KIF1Bβ 40aa(+)	KIF1Bβ 40aa(-)	XP_038965095.1, XP_038965102.1	249	Matsushita *et al.* (2009)
41	CALCA Calcitonin	CALCA CGRP	NP_001029124.1, NP_001029125.1	320	Leff *et al.* (1987)
42	Fox-1-TALVP	Fox-1-FAPY	NP_001346652.1, XP_036015842.1	170	Lee *et al.* (2009)
43	p21Ras	p19Ras	NP_005334.1, NP_789765.1	123	Jeong *et al.* (2006)
44	Igα	ΔIgα	NP_001774.1, NP_067612.1	33	Indraccolo *et al.* (2002)
45	CXCL12β	CXCL12γ	NP_000600.1, NP_001029058.1	156	Laguri *et al.* (2007)
46	Noxo1β	Noxo1γ	NP_751907.1, NP_751908.1	24	Ueyama *et al.* (2007)
47	KIF2A.1	KIF2A.3	NP_001361656.1, NP_001365866.1	332	Akkaya *et al.* (2021)
48	MYPT1 LZ(+)	MYPT1 LZ(-)	XP_015133610.1, NP_990454.1	164	Khatri *et al.* (2001)
49	CD40 type I	CD40 type II	NP_035741.2, NP_733804.1	34	Tone *et al.* (2000)
50	NOSTRINα	NOSTRINβ	NP_443178.2, NP_001034813.2	91	Mookerjee *et al.* (2007)

REFERENCES

Akkaya C, Atak D, Kamacioglu A, Akarlar BA, Guner G, Bayam E, Taskin AC, Ozlu N, Ince-Dunn G. **2021**. Roles of developmentally regulated KIF2A alternative isoforms in cortical neuron migration and differentiation. *Development* **148**: dev192674.

Astro V, Ramirez-Calderon G, Pennucci R, Caroli J, Saera-Vila A, Cardona-Londoño K, Forastieri C, Fiacco E, Maksoud F, Alowaysi M, *et al.* **2022**. Fine-tuned KDM1A alternative splicing regulates human cardiomyogenesis through an enzymatic-independent mechanism. *iScience* **25**: 104665.

Bianchi M, Amendola R, Federico R, Polticelli F, Mariottini P. **2005**. Two short protein domains are responsible for the nuclear localization of the mouse spermine oxidase µ isoform. *The FEBS Journal* **272**: 3052–3059.

Choi S, Lee HS, Cho N, Kim I, Cheon S, Park C, Kim E-M, Kim W, Kim KK. **2022**. RBFOX2-regulated TEAD1 alternative splicing plays a pivotal role in Hippo-YAP signaling. *Nucleic Acids Research*: gkac509.

Davis LH, Davis JQ, Bennett V. **1992**. Ankyrin regulation: an alternatively spliced segment of the regulatory domain functions as an intramolecular modulator. *Journal of Biological Chemistry* **267**: 18966–18972.

Fletcher RB, Baker JC, Harland RM. **2006**. FGF8 spliceforms mediate early mesoderm and posterior neural tissue formation in Xenopus. *Development* **133**: 1703–1714.

Frias MA, Thoreen CC, Jaffe JD, Schroder W, Sculley T, Carr SA, Sabatini DM. **2006**. mSin1 Is Necessary for Akt/PKB Phosphorylation, and Its Isoforms Define Three Distinct mTORC2s. *Current Biology* **16**: 1865–1870.

Gray NW, Kruchten AE, Chen J, McNiven MA. **2005**. A dynamin-3 spliced variant modulates the actin/cortactin-dependent morphogenesis of dendritic spines. *Journal of Cell Science* **118**: 1279–1290.

Gu P, Gong J, Shang Y, Wang F, Ruppell KT, Ma Z, Sheehan AE, Freeman MR, Xiang Y. **2019**. Polymodal Nociception in Drosophila Requires Alternative Splicing of TrpA1. *Current Biology* **29**: 3961–3973.

Hakre S, Tussie-Luna MI, Ashworth T, Novina CD, Settleman J, Sharp PA, Roy AL. **2006**. Opposing Functions of TFII-I Spliced Isoforms in Growth Factor-Induced Gene Expression. *Molecular Cell* **24**: 301–308.

Han SP, Friend LR, Carson JH, Korza G, Barbarese E, Maggipinto M, Hatfield JT, Rothnagel JA, Smith R. **2010**. Differential Subcellular Distributions and Trafficking Functions of hnRNP A2/B1 Spliceoforms. *Traffic* **11**: 886–898.

Iijima T, Wu K, Witte H, Hanno-Iijima Y, Glatter T, Richard S, Scheiffele P. **2011**. SAM68 Regulates Neuronal Activity-Dependent Alternative Splicing of Neurexin-1. *Cell* **147**: 1601–1614.

Indraccolo S, Minuzzo S, Zamarchi R, Calderazzo F, Piovan E, Amadori A. **2002**. Alternatively spliced forms of Igα and Igβ prevent B cell receptor expression on the cell surface. *European Journal of Immunology* **32**: 1530–1540.

Jeong M-H, Bae J, Kim W-H, Yoo S-M, Kim J-W, Song PI, Choi K-H. **2006**. p19ras Interacts with and Activates p73 by Involving the MDM2 Protein. *Journal of Biological Chemistry* **281**: 8707–8715.

Kang HC, Chae JH, Lee YH, Park M-A, Shin JH, Kim S-H, Ye S-K, Cho YS, Fiering S, Kim CG. **2005**. Erythroid Cell-Specific α-Globin Gene Regulation by the CP2 Transcription Factor Family. *Molecular and Cellular Biology* **25**: 6005–6020.

Khatri JJ, Joyce KM, Brozovich FV, Fisher SA. **2001**. Role of Myosin Phosphatase Isoforms in cGMP-mediated Smooth Muscle Relaxation. *Journal of Biological Chemistry* **276**: 37250–37257.

Ko W, Jung S-R, Kim K-W, Yeon J-H, Park C-G, Nam JH, Hille B, Suh B-C. **2020**. Allosteric modulation of alternatively spliced Ca2+-activated Cl− channels TMEM16A by PI(4,5)P2 and CaMKII. *Proceedings of the National Academy of Sciences* **117**: 30787–30798.

Kustatscher G, Hothorn M, Pugieux C, Scheffzek K, Ladurner AG. **2005**. Splicing regulates NAD metabolite binding to histone macroH2A. *Nature Structural & Molecular Biology* **12**: 624–625.

Laakso JM, Lewis JH, Shuman H, Ostap EM. **2010**. Control of myosin-I force sensing by alternative splicing. *Proceedings of the National Academy of Sciences* **107**: 698–702.

Laguri C, Sadir R, Rueda P, Baleux F, Gans P, Arenzana-Seisdedos F, Lortat-Jacob H. **2007**. The Novel CXCL12γ Isoform Encodes an Unstructured Cationic Domain Which Regulates Bioactivity and Interaction with Both Glycosaminoglycans and CXCR4. *PLOS ONE* **2**: e1110.

Larsson SH, Miyagawa K, Engelkamp D, Rassoulzadegan M, Ross A, Cuzin F, Hastie ND. **1995**. Subnuclear Localization of WT1 in Splicing or Transcription Factor Domains Is Regulated by Alternative Splicing. **81**: 391–401.

Lee H, Dean C, Isacoff E. **2010**. Alternative Splicing of Neuroligin Regulates the Rate of Presynaptic Differentiation. *Journal of Neuroscience* **30**: 11435–11446.

Lee J-A, Tang Z-Z, Black DL. **2009**. An inducible change in Fox-1/A2BP1 splicing modulates the alternative splicing of downstream neuronal target exons. *Genes & Development* **23**: 2284–2293.

Leff E, Evans M, Rosenfeld' G, Hughes H. **1987**. Splice Commitment Dictates Neuron-Specific Alternative RNA Processing in CalcitonidCGRP Gene Expression. **48**: 517–524.

Li D, Jin C, Yin C, Zhang Y, Pang B, Tian L, Han W, Ma D, Wang Y. **2007**. An alternative splice form of CMTM8 induces apoptosis. *The International Journal of Biochemistry & Cell Biology* **39**: 2107–2119.

Lin YS, Yasuda K, Assem M, Cline C, Barber J, Li C-W, Kholodovych V, Ai N, Chen JD, Welsh WJ, *et al.* **2009**. The Major Human Pregnane X Receptor (PXR) Splice Variant, PXR.2, Exhibits Significantly Diminished Ligand-Activated Transcriptional Regulation. *Drug Metabolism and Disposition* **37**: 1295–1304.

Liu Y-W, Surka MC, Schroeter T, Lukiyanchuk V, Schmid SL. **2008**. Isoform and Splice-Variant Specific Functions of Dynamin-2 Revealed by Analysis of Conditional Knock-Out Cells. *Molecular Biology of the Cell* **19**: 5347–5359.

Low R, Léguillette R, Lauzon A-M. **2006**. (+)Insert smooth muscle myosin heavy chain (SM-B): From single molecule to human. *The International Journal of Biochemistry & Cell Biology* **38**: 1862–1874.

Ma X, Kawamoto S, Uribe J, Adelstein RS. **2006**. Function of the Neuron-specific Alternatively Spliced Isoforms of Nonmuscle Myosin II-B during Mouse Brain Development. *Molecular Biology of the Cell* **17**: 2138–2149.

Malartre M, Short S, Sharpe C. **2006**. Xenopus embryos lacking specific isoforms of the corepressor SMRT develop abnormal heads. *Developmental Biology* **292**: 333–343.

Matsushita M, Yamamoto R, Mitsui K, Kanazawa H. **2009**. Altered Motor Activity of Alternative Splice Variants of the Mammalian Kinesin-3 Protein KIF1B. *Traffic* **10**: 1647–1654.

McCartney CE, McClafferty H, Huibant J-M, Rowan EG, Shipston MJ, Rowe ICM. **2005**. A cysteine-rich motif confers hypoxia sensitivity to mammalian large conductance voltage- and Ca-activated K (BK) channel α-subunits. *Proceedings of the National Academy of Sciences of the United States of America* **102**: 17870–17876.

Metcalf CE, Wassarman DA. **2006**. DNA Binding Properties of TAF1 Isoforms with Two AT-hooks*. *Journal of Biological Chemistry* **281**: 30015–30023.

Mookerjee RP, Wiesenthal A, Icking A, Hodges SJ, Davies NA, Schilling K, Sen S, Williams R, Novelli M, Müller-Esterl W, *et al.* **2007**. Increased Gene and Protein Expression of the Novel eNOS Regulatory Protein NOSTRIN and a Variant in Alcoholic Hepatitis. *Gastroenterology* **132**: 2533–2541.

Morris DH, Dubnau J, Park JH, Rawls JM, Jr. **2012**. Divergent Functions Through Alternative Splicing: The Drosophila CRMP Gene in Pyrimidine Metabolism, Brain, and Behavior. *Genetics* **191**: 1227–1238.

Mosbacher J, Schoepfer R, Monyer H, Burnashev N, Seeburg PH, Ruppersberg JP. **1994**. A Molecular Determinant for Submillisecond Desensitization in Glutamate Receptors. *Science* **266**: 1059–1062.

Péterfy M, Phan J, Reue K. **2005**. Alternatively Spliced Lipin Isoforms Exhibit Distinct Expression Pattern, Subcellular Localization, and Role in Adipogenesis. *Journal of Biological Chemistry* **280**: 32883–32889.

Sebastian S, Faralli H, Yao Z, Rakopoulos P, Palii C, Cao Y, Singh K, Liu Q-C, Chu A, Aziz A, *et al.* **2013**. Tissue-specific splicing of a ubiquitously expressed transcription factor is essential for muscle differentiation. *Genes & Development* **27**: 1247–1259.

Sundvall M, Peri L, Määttä JA, Tvorogov D, Paatero I, Savisalo M, Silvennoinen O, Yarden Y, Elenius K. **2007**. Differential nuclear localization and kinase activity of alternative ErbB4 intracellular domains. *Oncogene* **26**: 6905–6914.

Tang Y, Wang H, Wei B, Guo Y, Gu L, Yang Z, Zhang Q, Wu Y, Yuan Q, Zhao G, *et al.* **2015**. CUG-BP1 regulates RyR1 ASI alternative splicing in skeletal muscle atrophy. *Scientific Reports* **5**: 16083.

Tone M, Tone Y, Fairchild P, Wykes M, Waldmann H. **2000**. Regulation of CD40 function by its isoforms generated through alternative splicing. *PNAS* **98**: 1751–1756.

Ueyama T, Lekstrom K, Tsujibe S, Saito N, Leto TL. **2007**. Subcellular localization and function of alternatively spliced Noxo1 isoforms. *Free Radical Biology and Medicine* **42**: 180–190.

Unoki M, Shen JC, Zheng Z-M, Harris CC. **2006**. Novel Splice Variants of ING4 and Their Possible Roles in the Regulation of Cell Growth and Motility. *Journal of Biological Chemistry* **281**: 34677–34686.

Yang Y, Carstens RP. **2017**. Alternative splicing regulates distinct subcellular localization of Epithelial splicing regulatory protein 1 (Esrp1) isoforms. *Scientific Reports* **7**: 3848.

Zhu B, Gulick T. **2004**. Phosphorylation and Alternative Pre-mRNA Splicing Converge To Regulate Myocyte Enhancer Factor 2C Activity. *Molecular and Cellular Biology* **24**: 8264–8275.
